# ROS and Oxidative Response Systems in Plants Under Biotic and Abiotic Stresses: Revisiting the Crucial Role of Phosphite Triggered Plants Defense Response

**DOI:** 10.3389/fmicb.2021.631318

**Published:** 2021-07-01

**Authors:** Mohammad Aqa Mohammadi, Yan Cheng, Mohammad Aslam, Bello Hassan Jakada, Myat Hnin Wai, Kangzhuo Ye, Xiaoxue He, Tiantian Luo, Li Ye, Chunxing Dong, Bin Hu, S. V. G. N. Priyadarshani, Gefu Wang-Pruski, Yuan Qin

**Affiliations:** ^1^Joint FAFU-Dalhousie Lab, College of Horticulture, Fujian Agriculture and Forestry University, Fuzhou, China; ^2^State Key Laboratory of Ecological Pest Control for Fujian and Taiwan Crops, Fujian Provincial Key Laboratory of Haixia Applied Plant Systems Biology, College of Life Sciences, Center for Genomics and Biotechnology, Fujian Agriculture and Forestry University, Fuzhou, China; ^3^State Key Laboratory for Conservation and Utilization of Subtropical Agro-Bioresources, Guangxi Key Lab of Sugarcane Biology, College of Agriculture, Guangxi University, Nanning, China; ^4^Department of Horticulture, College of Agriculture, Alberoni University, Kohistan, Afghanistan; ^5^College of Plant Protection, Fujian Agriculture and Forestry University, Fuzhou, China; ^6^College of Agriculture, Fujian Agriculture and Forestry University, Fuzhou, China; ^7^National Education Commission, Nugegoda, Sri Lanka; ^8^Department of Plant, Food, and Environmental Sciences, Faculty of Agriculture, Dalhousie University, Truro, NS, Canada

**Keywords:** phosphite, oomycete, plant pathogens, biotic stress, abiotic stress, reactive oxygen species, oxidative stress, gene modification

## Abstract

Phosphite (Phi) is a chemical analog of orthophosphate [HPO_4_^3−^]. It is a systemic pesticide generally known to control the prevalence of oomycetes and soil-borne diseases such as *Phytophthora*, *Pythium*, and *Plasmopora* species. Phi can also control disease symptoms and the spread of pathogenic bacteria, fungi, and nematodes. Phi plays critical roles as a fungicide, pesticide, fertilizer, or biostimulator. Overall, Phi can alleviate the severity of the disease caused by oomycete, fungi, pathogenic bacteria, and nematodes (leave, stem, fruit, tuber, and root) in various plants (vegetables, fruits, crops, root/tuber crops, ornamental plants, and forests). Advance research in molecular, physiological, and biochemical approaches has approved the key role of Phi in enhancing crop growth, quantity, and quality of several plant species. Phi is chemically similar to orthophosphate, and inside the cells, it is likely to get involved in different features of phosphate metabolism in both plants and pathogens. In plants, a range of physiobiochemical alterations are induced by plant pathogen stress, which causes lowered photosynthesis activities, enzymatic activities, increased accumulation of reactive oxygen species (ROS), and modification in a large group of genes. To date, several attempts have been made to study plant-pathogen interactions with the intent to minimize the loss of crop productivity. Phi’s emerging function as a biostimulant in plants has boost plant yield and tolerance against various stress factors. This review discusses Phi-mediated biostimulant effects against biotic and abiotic stresses.

## Introduction

Pesticides and fungicides are used extensively during crop production, but these chemical agents are toxic to the environment and health. Therefore, the choice of environmentally friendly agents, which can be used as pesticides and fungicides, is essential. Salts of phosphorous acid, Phi (phosphite), are less harmful to the environment and have been used as a fungicide for over 40 years and still attract attention to be used as fungicides. Several chemical and biological compounds, known as resistance inducers, can persuade plant defense response commotion (Silva et al., 2011). Since the discovery of its protective effects in 1977, Phi (known as fostyl-Al Fosjet/Agri-fos) has been applied widely on various crops to control both disease symptoms and the spread of fungi. The active ingredient in these chemicals is Phi (H_2_PO_3_^2−^) (Guest et al., 1995). The chemical similarity of Phi to phosphate (Pi) indicates that the center of mechanism relies on disordering of some desperate aspects of Pi metabolism (McDonald et al., 2001). Still, no molecular template has been proposed which qualified to count for all the remarkable effects that Phi have on *Phytophthora* spp. and on a major set of plant hosts.

Phi are generally applied as a pesticide (fungicide), fertilizer, and biostimulator for plant defense responses (McDonald et al., 2001; Han et al., 2021). As a biostimulator, Phi has been proven to boost nutrient absorption and assimilation and enhance product quality and biotic and abiotic stress tolerance. Phi also improves plant growth, nutritional value, and production (Ávila et al., 2013). Besides, Phi is extensively applied to prevent pathogen spreads, and in many countries, it is registered as a pesticide (Wu et al., 2019). Although, Pi analogs applications are as fertilizers and its effects limited to phosphorus (P) nutrition. Phi effectively prevents plant pathogens caused by oomycetes, particularly *Peronospora*, *Plasmopara*, *Phytophthora*, and *Pythium* genus (Silva et al., 2011; Olivieri et al., 2012; Burra et al., 2014; Groves et al., 2015). Phi can directly inhibit oxidative phosphorylation of pathogen metabolism (Lobato et al., 2008; Norton and Swinton, 2018), and indirectly alleviate plant defense mechanisms, ultimately inhibiting the pathogenesis (Daniel and Guest, 2005). The pathogenesis-related genes increase resistance against diseases and increase soluble protein accumulations in *Arabidopsis*, potatoes, and tomatoes (Silva et al., 2011; Chandrasekaran et al., 2017). Besides, Phi increases systemic acquired resistance (SAR) signaling activities in various plants against oomycetes, for instance, *Phytophthora* (Lim et al., 2013), *Pythium* (King et al., 2010), and *Pseudoperonospora* species (Ramezani et al., 2017b).

**FIGURE 1:**
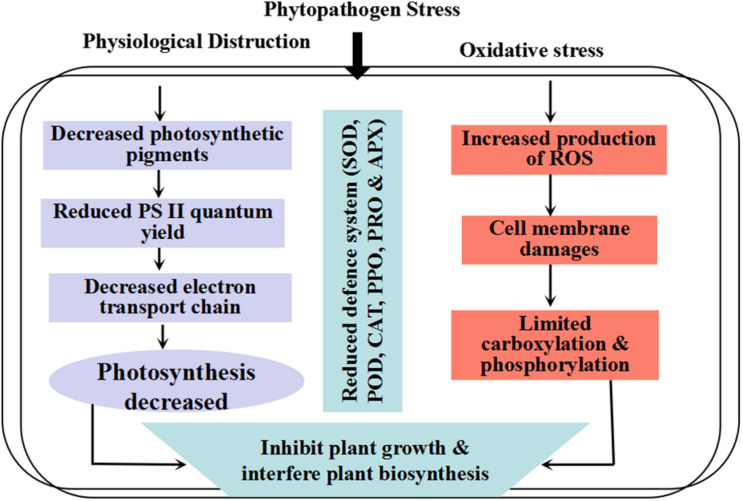
Schematic diagram interpreting the phytopathogen stress in plants.

*In planta*, the sensitivity to oxidative stress could result in the entire balance between factors that increase the production of antioxidants and cellular compounds. The interaction between plants and pathogens leads to physiochemical alteration in host plants. For example, photosynthesis reaction, respiration rate, and carbon uptake can be highly affected during pathogen infection (Raggi, 1978; Gonçalves et al., 2019). Damages to chlorophyll, photosystem II (PS II), and other components of the electron transport chain lead to a remarkable decrease in transport of photosynthetic electron chains. Therefore, considerable light energy is released as heat or fluorescence (Roháček et al., 2008). Previous reports suggest that several resistance inducers with low or minimum health risks can be used as an alternative to chemical fungicides (Thakur and Sohal, 2013; Tajik et al., 2019). During biotic and abiotic stresses, a high amount of reactive oxygen species (ROS) concentrations are induced, which could damage several introcellular macromolecules (Forouzanfar et al., 2016; Ramezani et al., 2018). These ROS with high oxidation rate cause damages to plant tissues, DNA mutation, cell organelle disordering, and decay of lipid, protein, and photosynthetic apparatus. It has been shown that a high level of resistance to the Phi-treated plants against biotic stresses is due to stimulation of antioxidant and defense enzymes in plant cells (Figure 1) (Oyarburo et al., 2015).

Plant oxidative response system, which includes non-enzymatic and enzymatic productions, have interfered with an aerobic process to neutralize oxidative cleavage due to ROS production (Figure 1). Protective enzymes include catalase (CAT), ascorbate peroxidase (APX), guaiacol peroxidase (GPX), superoxide dismutase (SOD), and polyphenol oxidase (PPO), while glutathione peroxidase (GR), ascorbate (AsA), proline (Pro), and carotenoids (Car) have non-enzymatic protection functions (Figure 5) (Jung et al., 2000; Lombardi and Sebastiani, 2005; Singh et al., 2009; Forouzanfar et al., 2016; Debnath et al., 2018; Ramezani et al., 2018). Like other biological tensions, fungal infections produce excessive free radicals, such as hydroxyl radical (^•^OH), hydrogen peroxide (H_2_O_2_), and superoxide radicals (^•^O_2_^–^), in cytosol, chloroplast, and mitochondria (Mofidnakhaei et al., 2016). It has been well documented that following antioxidant enzyme activation, plant tolerance to biotic stresses significantly increased (Lobato et al., 2011). The balance between ROS that resulted from loss to PS II and the alteration of oxidative stress can limit plant health and normal physiological process (Figure 1) (Dias et al., 2014). Changes in the expression of ROS molecules are a vital step in activation of plant response to phytopathogens. The antioxidant enzyme activities have been reported to prevent the release of oxidative molecules and permit cells to resist against the penetration of *Aphanomyces euteiches* and *Sclerotinia sclerotiorum* in plant tissues (Ali et al., 2006; Peluffo et al., 2010; Djébali et al., 2011).

### Invasion and Phytopathogenesis-Colonization of Plant Pathogens in Hosts

Vegetative hyphae can infect plant tissues in the same way as other infectious organelles (Hardham, 2007). Mycelia infection may occur due to root-to-root contact at the site (McCarren et al., 2005). The primary multiplication of zoospore pathogen infection is motile, and it is suggested that these have a radius of infection of 1–3 cm and can float for hours or even days (Hardham, 2007). The spores are often absorbing and encyst by tiny roots and are preferably located in the elongated area behind the meristems. The encystment may be caused by physical irritation, cold or increased calcium concentration, and the contents of the cytoplasmic vesicles secreted in the first few minutes after contact with the roots, forming a tape that binds to the plant cell wall (Hardham, 2007; Hardham and Blackman, 2018). The cyst then induces a germ tube that makes a hole into the cell wall outside or around the edge of the plant root cells by forming an aspersorium and then produces haustoria-like structures inside the cell that can absorb nutrients from the plant’s cell (Hardham, 2001). After getting into the plant cell, the phytopathogen obtains energy from glycolysis of plant rather than beta-oxidation (Torto-Alalibo et al., 2007) and grows asexually (Savidor et al., 2008).

Several pathogens that may be involved in plant inoculation have been isolated and identified as oomycetes. They consist of cell wall-destroying enzymes like endocellulase, β-1,3-glucanase (GLU), β-glucosidase, chitinase (CHI), pectinesterase, galactinase, and endopolygalacturonases (van West et al., 2003; Mohammadi et al., 2019). The majority of oomycetes generate extracellular RNAase, DNAse, phosphatases, lipases, amylases, proteases, and cellulase, which can all be associated with phytopathogenesis (Kamoun, 2006; Win et al., 2007). *Phytophthora* spp. also produces factors that enhance or suppress host defense by producing enzymes that chemically attack the host. Sequencing of *Phytophthora infestans* genome and comparison with other stramenopiles including diatoms and other omomycetes like *Phytophthora*, *Hyaloperonospora*, *Arabidopsidis*, and *Pythium ultimum* has revealed plenty of hydrolases. The ABC transports proteinase inhibitors and a combination of a wonderful family of 700 proteins with homology to the hormonal avirulence oomycete genes (Taylor et al., 2011). Plant defense responses are induced and stained by plant pathogens, to some extent, pathogenicity relies on protein-targeting patterns (Govers and Gijzen, 2006). New features of oomycete genomes contain the proliferation of genes that encode secretary effectors, plant cell wall-destroying enzymes in *Phytophthora*, and the over-representation of genes involved in photolytic degradation and signal transduction in *Pythium* spp. (Adhikari et al., 2013). Plant defense responsive cutters are induced and secreted by *Phytophthora* to some extent; it can be said that pathogenesis relies on protein-targeting motifs (Bengyella et al., 2019). A necrosis-inducing protein isolated from *P. cinnamon* facilitates the colonization of host tissues during the necrotrophic stage by stimulating plant cell death by facilitating nutrient uptake by pathogens (Martins et al., 2019). Effects of fungal and oomycete proteins have been shown to play an important role in apoplasts for adaptation, with recent advances in understanding the role of these proteins in escaping chitin-induced immunity (Rocafort et al., 2020). In this study, they also demonstrated the ability of apoplastic-affecting proteins to be incompatible with detection by different plant cell surface immune receptors and to use their effectiveness to rapidly detect protein-cell immune receptor interactions and apoplastic cells (Rocafort et al., 2020).

## Environmentally Friendly Agrochemical Ohosphite

Phi is an alkali metal salt of phosphoric acid (McDonald et al., 2001), and it should not be confused with Pi, which is derived from phosphoric acid (H_3_PO_4_). Phi salts contain a metal cation such as K^+^, Na^+^, or Mg^+^ and any of the following non-metal anions: Phi (PO_3_^3−^), hydrogen Phi (HPO_3_), or dihydrogen Phi (H_2_PO_3_). When phosphorous acid (H_3_PO_3_) reacts with water, it forms phosphonic acid, which is highly acidic and is neutralized by potassium hydroxide (KOH) to potassium dihydrogen Phi (KH_2_PO_3_) or dipotassium hydrogen Phi (K_2_HPO_3_). These two compounds are active ingredients in several fungicides, pesticides, and fertilizers (Landschoot and Cook, 2005). Inorganic H_3_PO_3_ salts can be found under various names in the literature. Phi (preferred here) and phosphonates are widely used, followed by the synonyms hydrogen phosphonates, ortho-Phi, phosphonic acid, and phosphorous acid (Hardy et al., 2001). They emphasized that the use of the term (Phi) clearly distinguishes inorganic salts of H_3_PO_3_ from phosphonates because the latter contains an organic group that binds to the P ion and is found in conventional chemical fungicides. The comparative advantage of Phi over phosphate (Pi) is that Phi can have direct toxic effects on fungi. The side effect of Phi is high-risk chemical toxicity when use at doses above 5 g/L or 36 kg/ha (Barrett et al., 2003). The utility of Phi in agriculture has been studied in relation to its influence on disease prevention than to be a plant fertilizer. The latter is possible when Phi is used to the soil and comes in contact with certain soil bacteria, which can oxidize them to Pi and absorb as P (Figure 6) (McDonald et al., 2001; Barrett et al., 2003). However, this process is long and may take 4 months; therefore, it has no actual significance (McDonald et al., 2001; Lovatt and Mikkelsen, 2006).

### The Chemistry of Phi

**FIGURE 2:**
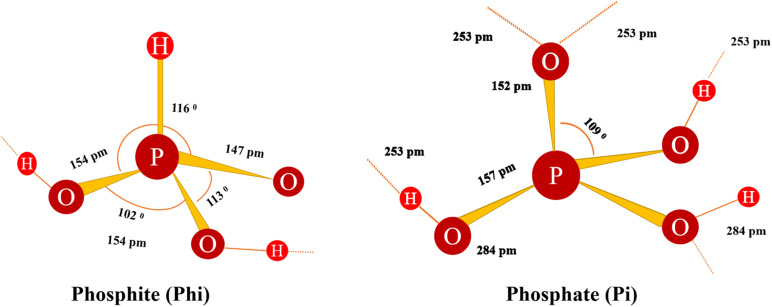
The structure of Phi shows the defined length and angle (Greenwood and Earnshaw, 2012). Broken shadow lines represent the potential H chains of other molecules.

In an aqueous solution, (H_3_PO_3_) is in equilibrium with tautomer H_3_PO_4_ (Equation 1) (Guthrie, 1979), but it turns into a quadrilateral form (Kraszewski and Stawinski, 2007). At neutral pH, the solution also contains a mixture of mono- and dianionic forms of phosphonate ions, known here as Phi (Figure 2; Equations 2 and 3). In Phi, P^+^ was oxidized, and the oxygen atom (O_2_^2+^ was replaced by a non-ionized hydrogen atom (Greenwood, 1984; Greenwood and Earnshaw, 1997).

Tautomeric equilibrium:

(1) $$P{\left(OH\right)}_{3}HPO{\left(OH\right)}_{2}$$

Phi dissociation:

(2) H3PO3→H2PO3+-H+pKa=1.3

(3) H2PO3→HPO3+2-H+pKa=6.7

### Phi as an Alternative Fungicide

Phi, a reduced form of Pi, is the oxyanion of phosphoric acid (H_3_PO_4_). When the Phi are dissolved in water, a strong acid form called phosphonic acid is produced. The addition of alkaline metal salts, such as K^+^, Ca^+^, Mg^+^, and Al^+^, preserves neutral pH; low pH itself is harmful to plant tissues. KOH is added to form the resulting solution, which is referred to as potassium mono or di-salt of phosphorous acid (KH_2_PO_3_/K_2_HPO_3_). Another solution, fosetyl-Al, was created by adding Al^+^ (Guest and Grant, 1991).

In the 1970s, Phi was discovered as an anti-fungal agent in the laboratory of Ron Polink in France. It is absorbed by membranes on the leaves and stems of plants and has good solubility and mobility. Phi is a phloem-mobile molecule that can be applied by spraying on leaves or injecting the trunk. Thus, foliar application of Phi based on fungicide was found with systemic properties to reduce the tumor and root diseases (Guest and Grant, 1991). Also, Phi fungicides are safe and environmentally friendly pesticides, according to the US Environmental Protection Agency (Lobato et al., 2017). Also, the fungicides resistance action committee (FRAC) classified Phi as grouped 33, as a low-risk fungicide^1^. Integrated pest management (IPM) is the safest and most effective way for pest control while minimizing pesticide use on plants. In the IPM program, Phi is an alternative product for promoting the reduction of toxic fungicides application (Mayton et al., 2008).

### Phi Mode of Actions

Phi’s mode of action in plants is divided into direct and indirect modes. The Phi direct mode of action is complicated (Massoud et al., 2012). Studies have shown that the direct mode of action depends on Phi concentration: higher levels of Phi have a direct inhibitory effect on various fungal and fungal-like organisms such as oomycetes and other species (Fo et al., 1990). The direct mode of action is related to the inhibition growth of zoospores and mycelia production (Cohen and Coffey, 1986; Wilkinson et al., 2001; Liu et al., 2020). After the addition of Phi in the culture plates, changes in gene expression levels were reported in *P. cinnamon* (King et al., 2010). Genes involved in the biosynthesis of the pathogen cell wall, like cellulose and glycan synthases, were inhibited in Phi (40 μg/ml) medium, and gene expression was not significantly altered in the medium containing relatively low (5 μg/ml) Phi. After adding Phi (40 μg/mL) for 4 days, about 70% of the *P. cinnamon* culture growth in the medium was inhibited. These results indicate that Phi directly inhibits the cell wall biosynthesis of pathogens and relatively low Phi concentration (5 μg/ml) may not be enough to directly prevent its growth (King et al., 2010).

In contrast to Phi’s direct mode of action, Phi’s low concentrations are associated with indirect action to boost plant defense responses as induced resistance (IR) (Coffey and Joseph, 1985; Jackson and Andrews, 2000). Phi-induced resistance (IR) studies suggest that Phi operates in more than one way in plants. Indirect actions of Phi are more useful to suppress pathogens than the direct effects (Daniel and Guest, 2005). Previous studies have revealed the biochemical changes that led to the suppression of pathogens (Guest and Grant, 1991; Jackson and Andrews, 2000; Bengtsson, 2013). Expression of many genes in plants was studied, and defense-related genes against *P. infestans* resistance were found to be upregulated (Eshraghi et al., 2011). The indirect molecular mechanism of Phi which inhibits *P. infestans* has been determined (Daniel and Guest, 2005; Massoud et al., 2012).

### Phi Uptake and Mobility Within Plants

Plants absorbed Phi easily and transport it in xylem and phloem as the same mechanism of Pi (Borza et al., 2017). Evidence suggests that Phi is actively mobile into plants and controls fungal development (Borza et al., 2014) or indirectly can become available to the plant as a P-source for plant nutrition after microbial oxidative reactions and improved the growth parameter in the host plants (Constán-Aguilar et al., 2014). The microorganisms in the soil and rhizosphere oxidize the Phi to Pi, which is easily absorbed by the roots of plants through the normal Pi channels. However, the process is slow and may take several months. Transportation, classification, biostimulant effects, and Phi fungicide effects were recently investigated in potato leaves using gas chromatography/time-of-flight mass spectrometry (GC-TOF-MS) (Wu et al., 2019). GC-TOF-MS showed that the Phi program modified some metabolite libraries. Phenylalanine (PAL) treatment is associated with higher levels of pathway accumulation of phenylpropane, chlorogenic acid, caffeic acid, and salicylic acid (SA). In Pi-starved tobacco cells, it was found that Phi competitively inhibits the influx of Pi through multiple transport mechanisms and accumulates in the cytoplasm, preferably vacuoles. In contrast, Phi-treated preloaded cells almost exclusively collected Phi in the vacuole (Danova-Alt et al., 2008). Depending on Pi status of the plants, the compartmentalization of Phi in either the cytosol or vacuole may explain why Phi is more effective in controlling oomycete diseases in low Pi soils and conversely requires more frequent application in plants grown on high Pi soils such as in potato fields (Wang-Pruski et al., 2010). Previously, it was reported that intracellular orthophosphate is acting as an inductor of adaptive response to stresses and is stimulated for better plant performance (Trejo-Téllez and Gómez-Merino, 2018).

### The Effects of Phi on Plant Chemistry

Phi seems to have a minor effect on tobacco cell processes and respiration (Danova-Alt et al., 2008). Unlike *Phytophthora* spp., treatment of tobacco cells with Phi did not reduce nucleoside triphosphates or glucose-6-phosphate, nor did it stimulate nucleoside diphosphates or pyrophosphate and adenylate pools. However, these results were obtained only in 20 h of incubation after Phi application. Whereas, Gent et al. (2020) documented that the occurrence of *Pseudoprosnopura humoli* isolates was at a higher level of sensitivity to fosetyl-Al and other Phi fungicides. Plant tissues contain pyrophosphate values in the range of 0.5–40 nmol/g of fresh weight (FW) (equal to 0.5–40 μM) (Heinonen, 2001). No measurable changes in pyrophosphate concentration were observed in *Brassica nigra* seedlings grown at 10 mM Phi concentration (Carswell et al., 1997). In comparison, the concentration of pyrophosphate in *Phytophthora palmivora* increases from the control concentration of 0.2 to 1.5 mm in the presence of Phi. In *Brassica*, *Arabidopsis thaliana* contained high pyrophosphate levels (Heinonen, 2001).

### Phi-Mediated Improvement of Plant Health

Depending on the concentrations applied, Phi has been documented to prevent Pi starvation response, alter physiology metabolism and growth, and enhance plant defenses against infection by various fungi (Table 1). Research on the effects of fosetyl-Al and Phi indicated that in general, high exogenous Phi dosage can be associated with a decline in plant normal growth and development (Carswell et al., 1997; Trejo-Téllez and Gómez-Merino, 2018). In addition, Phi reduced tiny root growth and decreased colonization of mycorrhizal fungi in onion (Sukarno et al., 1998). Phi application on healthy plants affects pollen fertility and seed germination of some annual species of *Eucalyptus* trees (Fairbanks et al., 2001). It seems that repeated use of Phi, in long term, can limit the diversity of plants in the ecosystem, and this effect can be lessened by proper timing of Phi programs. Avocado plant pollen tube germination and growth seemed to be sensitive to Phi treatment, although relatively high concentrations of Phi were used in these experiments and performed *in vitro* (Nartvaranant et al., 2004). Phi could have detrimental effects on plant growth and overall health, especially in P deficiency in plants (Carswell et al., 1997). Phi’s long-term effect relies on both type and time of plant inoculation with pathogens (Wilkinson et al., 2001). Injection of 50–100 g Phi/L protects *Banksias* and *Eucalyptus* plant infection for a minimum of 4 years, and the lesion is often present with callus (Shearer et al., 2006). However, 3 weeks after root inoculation with *P. cinnamon*, stem injection with Phi could not control *Escherichia marginata* growth. In *Banksia coccinea*, there is a U-shaped relationship between stem injection of Phi concentration and the effectiveness of Phi in preventing the spread of *Phytophthora cinnamomi*, belt formation, and growth retardation (Shearer et al., 2006; Shearer and Fairman, 2007). The results of Phi applied on citrus plants showed that Phi not only enhanced plant growth but also increased fruit nutrient and yield (Lovatt and Mikkelsen, 2006). Unlike orthophosphate; Phi was readily taken when citrus-deficient leaves were applied. Phi application improved various horticultural plant’s agronomical traits, for example, yield, growth, quality, and biomass stimulation (Gómez-Merino and Trejo-Téllez, 2015). Phi application on strawberry improves growth, root, and quality of fruits compared with control (Glinicki et al., 2010; Estrada-Ortiz et al., 2011). Pretreatment of isolated chickpea leaves with Phi and later on infection with *Phytophthora cryptogea* controlled growth of the pathogen. This effect was entirely back set by preapplication of Phi on plant leaves as a PAL pathway inhibitor (Saindrenan et al., 1990). Phi prevents stem canker in *Persea americana* and *Persea indica* caused by *Phytophthora citricola* (El-Hamalawi et al., 1995) and moldy core rot in apple fruits caused by the causal agent *Alternaria alternata* (Reuveni et al., 2003).

**TABLE 1:** Effects of Phi on enhancement on plant growth and physiological and biochemical attributes of different plants grown under different pathogen stressed.

Effect of Phi	Plant species, organism	References
Diseases suppression in plants	Incidence of diseases caused by *Phytophthora* spp., *Verticillium* spp., *Fusarium* spp., *Armillaria luteobubalina*, and *Plasmoparaviticola* is controlled by Phi treatment in host plants; Phi suppressed *P. infestans*, *F. solani*, and *R. solani* inoculation in *Solanum tuberosum*. Phi alleviated apical necrosis in *Mangifera indica* caused by *P. syringae.* Plants also revealed a delay in senescence after the Phi application.	Mohammadi et al. (2020); Borza et al. (2019); Ramezani et al. (2018); Lobato et al. (2008); and Torés Montosa (2006)
Phi-enhanced plants to Pi starvation	Phi enhanced the expression of Pi starvation-induced gene expression, improving the growth and defense system in tomato plants. Phi applied to Pi-sufficient white lupin elicited a complete Pi starvation response (PSR) by (enhancing root development and enzyme activity alleviation) whereas application of Phi to Pi-deficient plants enhanced PSR. Phi enhanced the PSR in Pi-deficient turnip.	Vinas et al. (2020); Gilbert et al. (2000); and Carswell et al. (1997)
Prevention of Pi uptake	Phi boosted Pi absorption by root protection in *Hakea sericea*.	Sousa et al. (2007)
Decrease pollen fertility and seed germination	Phi application decreased pollen fertility and seed germination in a concentration-dependent manner in several plants. Fosetyl-Al on field-treated apple, pear, and cherry orchards has been revealed to be very effective on flowering the year following treatment, and a positive effect was noticed both on the number and quality of flower buds.	Eshraghi et al. (2011) and Peyrard et al. (2015)
Improved phytoalexin and phenolic accumulation	Foliar application of Phi enhanced phytoalexin and phenolic stimulation by 12–24 h in potato slices after inoculation with *P. infestans*. Phi caused defense response and an accumulation of ROS and ethylene in pepper after pathogen challenged by *P. capsici.* Phi treatment reduced *Sclerotinia sclerotiorum* lesion development in common bean and was associated with triggered ROS and MDA accumulation and increase in host defense enzymes and limitation of pathogen growth.	Mohammadi et al. (2019); Liu et al. (2016); and Fagundes-Nacarath et al. (2018)
Improved hypersensitive reaction	Phi application increased high accumulation of plant defense response system on suppression of pathogens in apple after apple scab (*Venturia inaequalis*) after infection.	Felipini et al. (2016)
Prevention of the plant’s root elongation	Phi prevents root elongation under Pi stress in *A. thaliana*.	Varadarajan et al. (2002)
Inhibition of pathogen growth and development	Inhibited growth of the pathogen *in vitro* culture, enhanced vesicle formation within hypha, and inhibited zoosporogenesis in *Phytophthora* spp. Phi setback development in nematode species. Phi inhibited growth *in vitro* in *Verticillium* spp. Phi caused stunted growth under suboptimal also optimal Pi absorption in maize. Phi increased growth, fruit quality, and production in strawberries.	Belhaj (2017); Oka et al. (2007); Borza et al. (2019); Schroetter et al. (2006); and Estrada-Ortiz et al. (2011)
Phytotoxicity	The side effects of Phi are phytotoxic symptoms in several plant species including foliar necrosis, defoliation, abnormalities growth, chlorosis, reduced root growth, at high-dosage rates, and plant death.	Scott et al. (2016) and Shearer et al. (2006)

The similarity between the response of plants to Pi and the application of Phi, especially in long-term field trials, has led us to believe that Phi can act as a biological fertilizer or biostimulant. However, Thao and Yamakawa (2009) concluded that increasing plant health attribution due to Phi being a source of Pi is misleading, because the half-life of Phi to Pi is several months due to oxidation of soil microorganisms, and those test plants can become infected again with pathogenic oomycetes, which are highly decreased by Phi (Adams, 2004). Although the effects of Phi on plant health may vary, in most cases, the use of Pi fertilizers, in general, can increase plant performance and reduce disease susceptibility (Walters and Bingham, 2007). Phi application increased cucumber plant resistance to downy mildew by creating a rapid generation of antioxidants and a high reduction sensitivity to ROS production that programmed cell death (Ramezani et al., 2017a). Induction of wheat resistance to Russian aphid (*Diuraph isnoxia*) mediated by Phi was associated with increased PAL and enzymatic activity levels (Venter et al., 2014).

Systemic resistance induced against rust in *Vici faba* is shown by treating the leaf with 10 mM Phi (Walters and Bingham, 2007). This reaction is very close to the response observed in chestnut after its trunk is being injecting with different Phi dosages (Daniel and Guest, 2005). It was shown that plants treated with Phi after inoculation with *P. cinnamomi* responded quickly to the disease severity in injected trees than plants untreated with Phi. Recently, experiments were conducted in the greenhouse to study the effects of Ca-Phi (38 kg P/ha 1) on the soil characteristics and growth parameters of four kinds of soil green manures, three superphosphates (TSP), and control (no fertilization). After 8 weeks of planting, the soil biomass yield, Phi concentration in plant biomass, various soil P pools, and microbial biomass nutrients were measured. In addition, with the exception of lupin in the control (*Lupinus albus* L.), Phi has no negative effect on green manure performance (Fontana et al., 2021). The Phi concentration in plant biomass varies with species and soil types. The maximum concentration of mustard (*Brassica juncea* L.) in clay is about 400 mg Phi/kg. Compared with the control, the fertilization of TSP and Phi had similar effects on different P pools and microbial biomass nutrients (C, N, and P), although the response depended on the soil type. In sand, after adding Phi, the amount of P (P_*NCHO*__3_) much increased in TSP treatment, indicating that Phi is partially oxidized. Among clays with high P stabilization ability, Phi P_*NaHCO*__3_, which is higher than TSP, may promote the chemical P form due to different solubility (Fontana et al., 2021). The Phi-treated roots of avocado obtained protection from zoospores of *P. cinnamomi*, Phi enhanced the root resistance to pathogen invasion (van der Merwe et al., 1992). Besides, Phi application enhanced mycorrhizal formation in American chestnut and various other responses in plants (Table 1).

## Phi-Triggered Plant Defense Response Against Biotic Stresses

Phi-treated plants accumulate phytoalexin, phenolic, flavonoids, proline, hydrazine, H_2_O_2_, MDA, and SOD. Tobacco cultivar (cv.) NC2326 becomes resistant to *Phytophthora nicotianae* by rapidly inducing accumulation of sesquiterpenoid phytoalexin (antimicrobial substance) at the penetration site. After treating tobacco cultivar NC2326 with mevinolin, an inhibitor of sesquiterpene biosynthesis, the plants became sensitive to *P. nicotianae.* However, pretreatment with Phi in NC2326 followed by mevinolin exposure did not increase its resistance. Also, the application of Phi on tobacco cv. Hick is susceptible to *P. nicotianae*, and Phi applications increased phytoalexin levels, suggesting that Phi activated more than one defense response (Guest et al., 1995).

Phenolic compounds play a crucial role in providing physical and chemical barriers to pathogen growth at the site of infection. This compound is produced by the phenylpropane pathway and is derived from the amino acids phenylalanine and tyrosine (Candela et al., 1995; Jiang et al., 2019). To study the phenylpropane pathway for Phi protection (Jackson and Andrews, 2000), the activities of two enzymes involved in the phenylpropanoid pathways, 4-coumarate coenzyme A ligase (4-CL) and cinnamyl alcohol dehydrogenase (CAD), and concentration of soluble phenolic and Phi were measured. They found that when the Phi concentration in the root is low, it interacts with the pathogen at the entry site to stimulate host defense enzymes. At high concentrations of Phi, it can directly affect the pathogen before it can communicate with the host to prevent it from growing, and the host defense remains unchanged. In potato *P. infestans* pathosystems, Phi-treated potato leaves had an increased amount of phenolic and phytoalexin after pathogen infection. The antioxidant enzyme compounds after Phi treatments were also increased (Machinandiarena et al., 2012; Mohammadi et al., 2019).

In addition to the accumulation of phytoalexins and phenolic, after KPhi application on potatoes sensitive to *P. infestans* increased antioxidant enzyme activities levels in potato tubers (Mohammadi et al., 2019). Under the light microscope, SOD release, rapid cytoplasmic aggregation, and nuclear migration were observed in Phi-treated tobacco against *P. nicotianae* and Phi-treated *Arabidopsis* against *P. palmivora* (Guest and Grant, 1991; Daniel and Guest, 2005). Recently, Phi-treated *Arabidopsis* leaves showed faster and intense callus deposition and H_2_O_2_ production than untreated leaves after infection with *P. cinnamomi* (Eshraghi et al., 2011). Olivieri et al. (2012) reported increased pectin in the cortex of tubers obtained from Phi-treated plants.

Current knowledge about plant defense responses has been extensively reviewed (Boller and He, 2009; Gimenez-Ibanez et al., 2019). The defensive response of plants to pathogens can be divided into two categories: the initial response to initial resistance that occurs within minutes and the response to resistance to specific host-pathogen genes that lasts for several days. To infect plants, the pathogen must first penetrate the physical barrier of the cell wall and neutralize any pre-prepared antimicrobial compounds, such as phytoalexins. The next hurdle is preventing plants from recognizing pathogen-related molecular patterns (PAMP) by non-self-aware systems (Nejat and Mantri, 2017). Examples of PAMP are fungal chitin and bacterial flagella; PAMP forms a non-specific basic immune response, including ion penetration, protein phosphorylation cascade including mitogen-activated protein kinase (MAPK) and ROS, and signaling molecules such as salicylic acid (SA) and jasmonic acid (JA) ethylene (ET) production. Induction of genes related to defense (such as hydrolase), phytoalexin biosynthesis, callus production, cell wall expansion, and possibly programmed cell death (Boller and He, 2009; Mohammadi et al., 2020).

In case of attack by pathogens, germinating plants show rapid defense responses. Natural or synthetic compounds can be used to chemically induce the onset of plant defense, and plants with previous experience of pathogen contamination show an initial defense response (Martinez-Medina et al., 2016). Saffron leaves that have been pretreated with different concentrations of SA under greenhouse conditions accumulated high levels of phenolics and flavonoids; activity and gene expression of PAL enzymes increased profiled proteins before and after Phi treatments in potato leaves (Lim et al., 2013; Tajik et al., 2019). These findings strongly demonstrate that priming plant leaves for 3 days induced the production of a large number of defense proteins against *P. infestans.*

**FIGURE 3:**
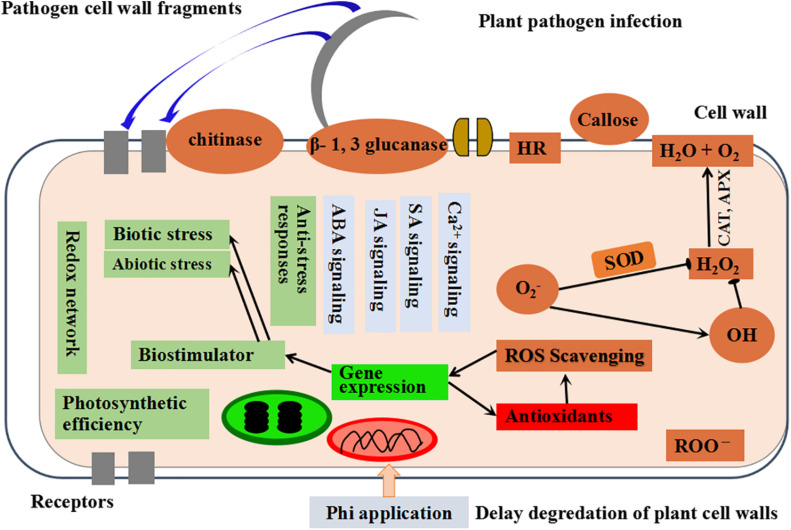
The schematic presentation showing the mechanism of Phi-induced activities in plants modified from Mohammadi et al. (2020).

Several crop species that had been sprayed with Phi and inoculated with different pathogens showed protection functions to the plants; such protection is related to the rapidly increased amounts of phytoalexin and ROS than untreated, infected plants (Ramezani et al., 2017a; Borza et al., 2019; Mohammadi et al., 2019). Cucumber plants infected with *Pythium ultimum* and treated with Phi-evidenced induction of antioxidant enzymes decreased ROS and alleviated damping-off symptoms (Mofidnakhaei et al., 2016). Saindrenan et al. (1990) reported that the resistance induced by Phi treatment to the isolated leaves of cowpea after inoculation with *P. cryptogea* could be setback by pre-application of the leaves with aminoxyacetate (AoA) and PAL ammonialyase inhibitor in the phenylpropanoid pathway. This suggests that phytoalexin biosynthesis is responsible for mediating the effects of Phi. Several changes in the host-pathogen interface have been observed after the treatment of plants with Phi. For example, Jiang and Hartung (2008) showed that Phi increases in the development of electron deposits are formed in uninfected neighboring cells. However, in the extraction of enhanced defense response, the indispensable factor is the presence of Phi and pathogens (Figure 3) (Jackson et al., 2000).

Pathogens also produce defense-inducing effects, such as viral gene product (AVR) proteins, lecithin, and elicitors. Pathogen molecules can be specifically identified by plant host resistance proteins (R proteins), which can determine the resistance between specific pathogens and hosts (Cui et al., 2015). The detection of pathogen AVR protein through plant dependence and R protein is more complicated than the simple interaction proposed by the gene-gene concept and can be described more accurately by the guard hypothesis (Jones and Dangl, 2006) or the decoy model (van der Hoorn and Kamoun, 2008). The guard hypothesis says that plant R-proteins (protectors) are associated with endogenous host proteins that are common-target proteins for pathogens. The interaction of effective pathogen proteins with host proteins changes their structure, which is then recognized by protective proteins. R-protein activation is highly regulated in plants because it ultimately leads to programmed cell death. However, early detection is important to successfully defend against plants. Any model of the effect of Phi on plants requires an increase in defense responses in the treated plants. However, if Phi has a direct effect on pathogens, it is essential. The low Phi activity *in vitro* indicates that the plant immune response is involved to a certain extent, so it is concluded that plants without a dynamic immune response are not protected by Phi (Arfaoui et al., 2020). Based on a 2-year field experiment, Borza et al. (2019) documented that a mixed-mode of Phi affects both plant defense and pathogen growth *in vitro.* However, the results were consistent with the site of Phi operation in *Verticillium* spp.; these studies indicate that plant defense plays a vital role in preventing fungi development.

### Phi Mediated Improvement in Alleviation of Adverse Effects of ROS in Plants After Being Challenged by Plant Pathogens

**FIGURE 4:**
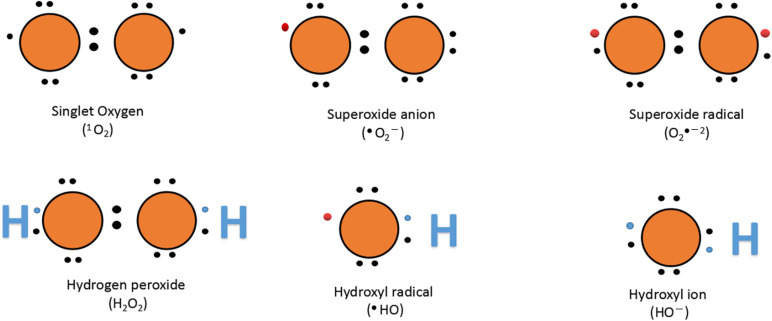
Lewis dot structure of oxygen and ROS. The name and chemical formula are given below each structure; bullets (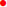) represent an unpaired electron.

Atmospheric oxygen (O_2_) is a free molecule, and triple oxygen (3O_2_) exists on earth. The two electrons of parallel and unpaired rotation have the same number of rotations and lose their reactivity. However, the extra energy from other biochemical reactions, electron transfer chains, ultraviolet B, and ionizing radiation helps to release 3O^2^ from the limitation of rotation and convert it to ROS (Figure 4) (Mailloux, 2016).

Many reports have shown that during several stresses, a large amount of ROS is produced that can damage intracellular macromolecules (Forouzanfar et al., 2016; Ramezani et al., 2018). The balance between ROS due to PS II damages and antioxidant enzyme activity can determine plant safety (Dias et al., 2014). Preliminary reports indicated that Phi induces high resistance on pretreated plants to pathogenic stresses and is mainly related to the high accumulation of defense enzymes in host plant (Oyarburo et al., 2015).

Plant antioxidants and oxidative enzyme production are enhanced by aerobic change, balancing the oxidative damage caused by ROS. Defense enzymes including CAT, APX, SOD, and various molecules including GR, Prl, AsA, and Car have non-enzymatic protective function (Jung et al., 2000; Lombardi and Sebastiani, 2005; Singh et al., 2009; Forouzanfar et al., 2016; Debnath et al., 2018; Ramezani et al., 2018). Similar to other biological stresses, fungal infections cause the excess production of free radicals, such as hydroxyl radicals (OH^•^), H_2_O_2_, and SOD production (Mofidnakhaei et al., 2016). It is well described that after activation of antioxidant enzymes, the plant’s tolerance to biotic stress significantly increased (Lobato et al., 2011). Phi has a significant influence on various enzymatic processes (CAT, POD, SOD, APX, PPO, and GPX) and non-enzymatic processes (phytoalexins, phenolics, flavonoids, and anthocyanins), which support the fight in the overproduction of ROS caused by pathogens in stimulating the integration of plant cells (Figure 3). O_2_ acts as an electron receptor with subsequent accumulation of ROS such as singlet oxygen (^1^O_2_), hydroxyl radical (OH^–^), superoxide radical (^•^O_2_^–2^), and H_2_O_2_ under stressful conditions. Low concentrations of Phi (0.005%) have been shown to help protect plants against ROS-induced oxidative damage; however, higher concentrations of Phi act as a per oxidant, causing ROS stimulation and oxidative stresses (Liu et al., 2016).

Many researchers have described Phi for increasing ROS inhibitory activity, decreasing MDA concentration, membrane damage, and triggered gene expression (Mofidnakhaei et al., 2016; Feldman et al., 2020). Besides, reduced production of H_2_O_2_ under Phi treatment has also been confirmed (Novaes et al., 2019). Under fungi stress, lowered H_2_O_2_ contents were observed in Phi-treated soybean (Batista et al., 2020). Meanwhile, plants treated by Phi showed fewer amounts of MDA under heat stress, indicating that Phi is critical in reducing lipid peroxidation by increasing antioxidant enzymes and protecting the membrane structures of potato seedlings (Xi et al., 2020), *Cucumis sativus* L. (Ramezani et al., 2017a), and *Glycine max* L. (Batista et al., 2020). Also, it was observed that the production of lipid MDA decreases with increasing Phi concentration under pathogen stress (Mohammadi et al., 2020). The comprehensive effect of MDA on plant cells is to reduce membrane fluidity to increase membrane leakage and to prevent damage to proteins, enzymes, and membrane in ion channels (Saed-Moucheshi et al., 2014). Appropriate concentrations of Phi are useful for reducing lipidoxygenase overexpression to maintain the formation of fatty acids in addition to the reduced generation of ROS, which is driven by the regulation of antioxidant systems (Ahanger and Agarwal, 2017).

### Phi Mediated Improved Plant Resistance by Modulation of Enzymatic and Non-enzymatic Antioxidants

**FIGURE 5:**
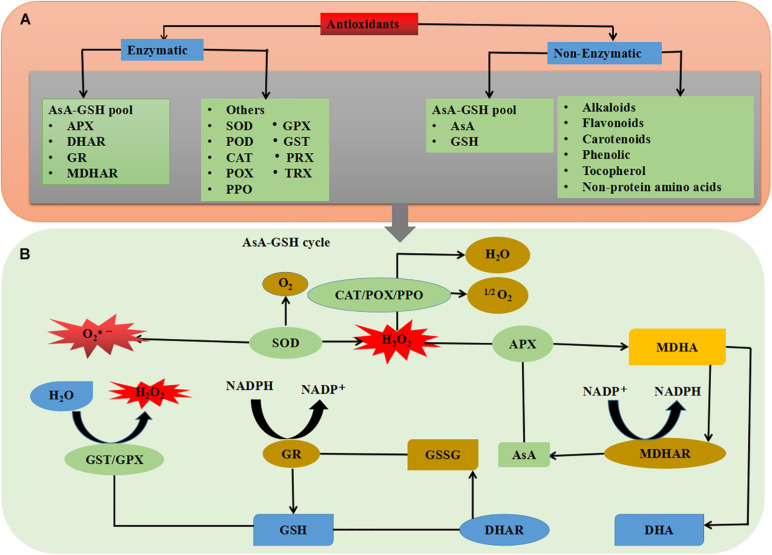
Schematic overview of plant antioxidant system; **(A)** types of antioxidants and **(B)** combined mechanism of enzymatic and non-enzymatic antioxidants. See the text for more information.

The antioxidant defense system including both non-enzymatic antioxidants and some antioxidant enzymes consists of lower molecular weight (Hasanuzzaman et al., 2019). Non-enzymatic antioxidants like AsA, reduced gluthione (GSH), α-tocopherol, phenolic, flavonoids, alkaloids, and non-protein amino acids work in a coordinated path with antioxidant enzymes such as SOD, CAT, POD, PPO, APX, monodehydroascorbate reductase (MDHAR), dehydroascorbate reductase (DHAR), GR, GPX, gluthione-*S*-transferase (GST), thioredoxin (TRX), and peroxireductinase (PRX) to control ROS production (Figure 5) (Nath et al., 2018; Laxa et al., 2019). *In planta*, SOD is directly related to the stress that initiates the first line of defense, by converting O_2_^–^ to H_2_O_2_ (Table 2) (Biczak, 2016; Luis et al., 2018). Produced H_2_O_2_ can be further converted to H_2_O by CAT, APX, and GPX enzymes or catalyzed by the AsA-GSH cycle. In a plant cell, the AsA-GSH is the main antioxidant defense pathway in H_2_O_2_ detoxification, which consists of non-enzymatic antioxidants AsA and GSH, as well as the four important enzymes APX, MDHAR, DHAR, and GR. In antioxidant defense system, the AsA-GSH cycle plays a key role in minimizing H_2_O_2_ homeostasis and redox (Hasanuzzaman et al., 2019). Furthermore, the GPX-GST is also an essential enzyme for H_2_O_2_ and xenobiotic detoxification (Figure 5) (Hasanuzzaman et al., 2018). Among the non-enzymatic antioxidants, AsA and GSH are the most abundant soluble antioxidants in higher plants, which play an important role as electron donors, and capture ROS directly in the AsA-GSH cycle (Hasanuzzaman et al., 2019). Furthermore, β-carotene reacts with ^•^OH, ^•^O_2_^–^ and ROO• radicals which cause a decrease in ROS production in plant cells (Figure 5) (Kapoor et al., 2019).

**TABLE 2:** Phosphite mitigates pathogen stress-induced oxidative damage by changes in different antioxidant enzyme activities in several plant species.

Plant species	Diseases	Causal agent	Method application of	Antioxidant activity	References
*Avena sativa* L.	White mold	*Sclerotinia sclerotiorum*	Foliar application	CHI ↑ GLU ↓ PPO ↓ SOD ↑ APX ↑	Fagundes-Nacarath et al. (2018)
Common bean	Without inoculation	*Normal condition*	Root application	CAT ↑ POD ↑	Ávila et al. (2013)
*Cucumis sativus* L.	Damping off	*Pythium ultimum* var.	Foliar spray	SOD ↑ POD ↑ CAT ↑	Mofidnakhaei et al. (2016)
*Cucumis sativus* L.	Downy mildew	*Pseudoperonospora cubensis*	Foliar spray	CHI ↑ GLU ↑ PPO ↓	Ramezani et al. (2018)
*Glycine max* L.	White mold	*Sclerotinia sclerotiorum*	Foliar spray	CHI ↑ GLU ↑ SOD ↓ POD ↓ CAT ↑ PAL ↑	Novaes et al. (2019)
*Malus pumila*	Apple scab	*Venturia inaequalis*	Foliar spray	POD ↑ GLU ↓	Felipini et al. (2016)
*Solanum tuberosum* L.	Late blight	*Phytophthora infestans*	Foliar application	SOD ↑ POD ↑ CAT ↑ APX ↑	Mohammadi et al. (2020)

Plants produce a set of antioxidant enzymes after exposure to various stresses, and it is interesting to note that the use of Phi has been shown to increase the antioxidant enzyme activities and decrease the ROS overproduction to cope with stress (Mohammadi et al., 2020; Xi et al., 2020). In pathogen stress, ROS can be detoxifying by antioxidant compounds (Table 2). Antioxidant enzymes, such as SOD, POD, APX, CAT, PPO, CHI, and GLU, are believed to positively react to Phi application in order to induce pathogen tolerance in plants (Mohammadi et al., 2019; Novaes et al., 2019). The researchers hypothesized that increasing the level of Phi-mediated antioxidant defense is one of the key mechanisms which can protect the plant from oxidative stress stimulated by the phytopathogens (Machinandiarena et al., 2012). SOD, APX, and CAT activities were significantly enhanced by Phi treatment in potato and cucumber seedlings under *P. infestans* and *Pseudoperonospora cubensis* stress, respectively (Mofidnakhaei et al., 2016; Ramezani et al., 2018; Mohammadi et al., 2020). In another study, the accumulation of antioxidants in cucumber due to Phi mediated increased levels of SOD, POD, APX, and CAT activities in plants (Mofidnakhaei et al., 2016). An increase in activities of the SOD, CAT, POD, APX, and PAL enzymes is observed in different crops like potato, common bean, *Avena sativa* L., and *Solanum lycopersicum* L. (Fagundes-Nacarath et al., 2018; Mohammadi et al., 2019), as seen in Table 2. One of the first changes after Phi application on *P. palmivora* mycelium treatment is the size of adenylate nucleotides (Griffith et al., 1993). Adenylate is involved in the synthesis of purines, pyrimidines, aromatic amino acids, nicotinamide adenine dinucleotides (NAD^+^), and nicotinamide adenine dinucleotide phosphate (NADP^+^) (Nelson et al., 2008). The evidence of the increases in enzyme activity observed in the body can be explained as a secondary effect of Phi. This is because transcriptional induction is a homeostatic reaction that limits the inhibitory effect of Phi on the enzyme. The organism tries to increase the metabolism through a pathway (Nelson et al., 2008). Besides, the increase in SOD accumulation is caused by the rapid conversion of ^•^O_2_^–^ to H_2_O_2_, which is produced in the chloroplast electron transport chain of the mitochondria. Evolving H_2_O_2_ was neutralized by CAT in the cytoplasm or by APX in the ASA-GSH pathway. In addition, increased SOD activity in Phi-treated seedlings, following optimum defense of chloroplast yield, altered the likelihood of ^•^OH^–^ synthesis (Nouet et al., 2011). Inhibition of H_2_O_2_ and MDA to water and lipid alcohol is performed by two important enzymes: GSH-POX and GR (Lim, 2012). GSH-PX is considered a vital enzyme, which is strongly activated by Phi in different plants under different environmental stresses (Xi et al., 2020). Increasing the activity of antioxidants reduces the levels of H_2_O_2_ and MDA and improves pepper and cucumber plants by overcoming oxidative damage stimulated by ROS under pathogen stress (Liu et al., 2016; Mofidnakhaei et al., 2016). Phi supplementation in citrus byproducts regulates the antioxidant system by increasing the activity of SOD, CAT, and APX after four treatments on coffee seedlings. In addition, increasing the antioxidant content continuously evolves in defense of the PS electron transport chain by maintaining better levels of the NADP + and inhibiting the composition of toxic radicals (Fernandes et al., 2014). These results show that proper use of Phi can be useful to improve the antioxidant defense mechanism of plants under pathogen stress.

### Phi Suppresses Adverse Effects of Oomycete Pathogens

The use of Phi-based fungicides can strengthen plants for a rapid and vigorous defense response to many diseases, fungi, and oomycetes, such as the *genera Phytophthora*, *Fusarium*, and *Rhizoctonia* (Machinandiarena et al., 2012; Alexandersson et al., 2016). Johnson et al. (2004) evaluated the effectiveness of the commercial prescription of Phi Phostrol (containing 53.6% of sodium and potassium salts of Phi and ammonium) to control tuber rot caused by *P. infestans* and *Phytophthora erythroseptica*. The mean prevalence and intensity of late potato disease and facial rot in tubers obtained from two treatments at 7.49 kg/ha were lower than control tubers. Miller et al. (2006) reported that the application of twice phostrol (8 pints/acre) for late blight and three times (10 pints/acre) for pink rot was significant to protect tubers from these diseases. Moreover, postharvest application of 14% phostrol for late blight and pink rot suppression after 77 days in storage was examined; Phi was able to control 90% of late blight and 61% of pink rot symptoms when compared with untreated potato tubers (Şandor and Opruţa, 2012).

Seed tubers and potato leaves pretreated with Ca and K salts of phosphorous acid were examined (Lobato et al., 2008). With a ratio of 1% (V/V) as a commercial product equal to 3 L/ha, Phi provided tubers with a protective level from *P. infestans*, while excellent protection of foliage was provided in four application rates of 2% of the product. In contrast, systemic fungicides, such as chlorothalonil (Bravo) or mancozeb (Dithane or Manzate), were applied on potato plants in combination with Phi during the growing season in Canada (Wang-Pruski et al., 2010). In a greenhouse study, foliar sprays of 1% KH_2_PO_3_ three times with an interval of 15 days significantly reduced the severity of late blight in potato tubers of two Chinese potato cultivars Xinjia No. 2 and Zhongshu No. 3, 50% and 40%, respectively, on the tubers of untreated potato plant (Mohammadi et al., 2019). In another experiment by Wang-Pruski et al. (2010), a 3-year field trial was conducted to evaluate Phi’s efficacy (Confine^TM^ containing 45.8% mono- and di-potassium salts of phosphorous acid) on potato late blight of Prince Edward Island, Canada. Analysis of the severity of the disease indicated that the leaves of the potato plants were pretreated five times in 2007 and 2008 at a ratio of 58 L product/ha, and the disease was significantly less than that of the untreated plants. The combined use of Phi and chlorothalonil provided better disease inhibition compared with the use of Phi or chlorothalonil alone. Also, by using Phi alternately with Bravo, the usage of Bravo was decreased by 50%. The late blight pressure was high in both field seasons, but Phi alone or in combination with Bravo provided complete control of late blight (Wang-Pruski et al., 2010).

In addition, Phi is effective as protectant fungicides against Dieback disease in indigenous plant species in Australia, like *Banksia brownie* (Hardy et al., 2001; Barrett et al., 2003), Brown rot in two citrus plants, *Citrus volkameriana* and *Citruslimonia* (Förster et al., 1998; Oren and Yogev, 2002). The incidence of *Phytophthora capsici* in tomato and pepper plants grown hydroponically by adding Phi was significantly reduced. On the other hand, foliar application of Phi in strawberry (*Fragaria ananassa*) plants effectively protects fruits against *Phytophthora cactorum* leather rot for up to 7 days and also has a therapeutic activity of at least 36 h (Rebollar-Alviter et al., 2007). Likewise, foliar application of Phi decreased the prevalence of *P. infestans* and *P. erythroseptica*, although Phi did not affect the suppression of *Pythium ultimate* (Johnson et al., 2004). By combining foliar and postharvest treatment of Phi, potato tubers were effectively controlled against *P. erythroseptica*, which induced pink rot during storage (Taylor et al., 2011). Similarly, the postharvest application of Phi effectively controlled the spread of potato tubers during storage (Lobato et al., 2011; Mohammadi et al., 2019). Also, when the leaves were treated with Phi, the susceptibility of the tubers to *P. infestans* was reduced significantly (Liljeroth et al., 2016). Whereas, the use of Phi on harvested tubers effectively controlled oomycete during storage (Lobato et al., 2008). Moreover, combining Phi with a wide range of non-systemic fungicides like chlorothalonil showed the most effective control against potato late blight disease (Liljeroth et al., 2016). In vinca, 0.5 mM foliar application of Phi in 3–6 days meantime highly protects plants against *P. nicotianae*, as performed by spraying 3 g/L Aliette at 14-day intervals (Banko and Hong, 2004). In *Banksia brownii*, Phi gave high resistance to *P. cinnamomi* in the initial infection stage (Barrett et al., 2003). In *Banksia grandis*, *B. coccinea*, and *Eucalyptus marginata*, Phi’s trunk injection gave reasonable control of *P. cinnamomi* and provides efficient control of the endangered native flora against this pathogen (Shearer et al., 2006). Also, Phi used on leaves alleviate death of four *P. cinnamomi*-infected *Banksia* spp. (Shearer and Fairman, 2007; Shearer and Crane, 2009). In the species of *Banksia* (Shearer and Crane, 2012), the changes in genotype observed follow the Phi treatment to control this oomycete, and (Eshraghi et al., 2014) Phi has been reported to induce resistance to those pathogen in *Arabidopsis*, which also has been confirmed in *Banksia grandis* and *Eucalyptus marginata* plant as seen in Table 3 (Scott et al., 2015).

**TABLE 3:** Suppression of oomycetes/*Phytophthora* spp. diseases by different sources of phosphite on several plant species.

Plant	Disease	Causal agent	Experimental details	Phosphite source (dosage)	References
Avocad o	Root rot	*P. cinnamomi*	Trunk injection	Potassium phosphite	Mcleod et al. (2018)
Banksia	*P. cinnamomi*	*P. cinnamomi*	Glasshouse	Mono-dipotassium phosphite	Barrett et al. (2003)
Citrus	Brown rot	*P. citrophthor a*	Pot culture (soil and foliar application)	Potassium phosphite	Oren and Yogev (2002)
Orange	Brown rot	*P. citrophthora*	Soilless media culture	Potassium phosphite	Orbović et al. (2008)
Papaya	Fruit rot	*P. palmivora*	Growth	Potassium phosphite	Smillie et al. (1989)
Pepper	Crown rot	*P. capsici*	Chamber (pot culture) hydroponic culture	Phosphorous acid	Förster et al. (1998)
Potato	Pink rot	*P. erythroseptica*	Field trial	Phosphorous acid	Taylor et al. (2011)
Potato	Late blight	*P. infestans*	Pot culture Peat, perlite, and vermiculite	Potassium phosphite	Mohammadi et al. (2020)
Strawberry	Leather rot	*P. cactorum*	Pot culture (peat, steam disinfected soil and sand)	Phosphorous acid	Rebollar-Alviter et al. (2007)
Tobacco	Black shank	*P. nicotianae*	Growth chamber (pot culture)	Potassium phosphite	Guest et al. (1995)
Vinca	Shoot blight	*P. nicotianae*	Containers (pine bark medium)	Potassium phosphonate	Banko and Hong (2004)

### General Characteristics and Economic Values of Oomycetes

According to Dick’s classification in 2001, the oomycetes belong to the Chromista/Stramenopile kingdom and are commonly referred to as water mold and downy mildew. Miscellaneous algae (including brown algae and golden algae) originated from Stramenopiles. Sparrow reported the taxonomic classification of oomycetes in 1960 and 1976 and Dick’s scientific work and research in 2001 (Birch and Whisson, 2001). They divided all oomycetes into two main groups, the first group containing water molds (Eurychasmales, Leptomitales, and Saprolegniales) and the second group belonging to the Peronosporalean order (Rhipidiales, Pythiales and Peronosporales). Rhipidiales and Albuginea belong to the Peronosporalean branch like Peronosporales (Birch et al., 2012). According to Blum et al. (2012), the cell wall of the true fungi consists of chitin, but the oomycete cell wall is formed of cellulose and β-1,3-glucan. Therefore, it is classified as pseudo-fungi. However, due to some unique biological characteristics, the difference between oomycetes and other eukaryotic microorganisms is still in discussion. The vegetative growth of oomycete*s* in the filaments produces mycelium and can go through sexual and asexual spore replications. Oomycetes contain tubular forms of mitochondria to synthesize lysine (Vogel et al., 1970). Zoospores are formed by the cleavage of cytoplasmic membranes of asexual spores (Latijnhouwers et al., 2003). Zoospores include two flagella, one’s tinsel anterior flagellum, and another whiplash flagellum. It contributes to the movement of mononuclear nucleated cells (Walker and van West, 2007). Oomycetes belong to a diploid trophic stage, but genetic recombination was not known in homologous diploid cells of other fungi. Due to the lack of homologous recombination, few species of genus *Phytophthora* have been reported, such as *P. infestans*, *Phytophthora ramorum*, and *Phytophthora sojae* (Tyler, 2007; Schornack et al., 2009). The genome size of oomycetes varies from 18 to 37 Mb (Judelson, 2012). Molecular research revealed that the oomycete genome consists of repetitive sequences, and some genes such as *Cytb* are much conserved among *Phytophthora* spp. (Mao and Tyler, 1996; Lamour et al., 2007). However, many researchers are still working on the genome of *Phytophthora* spp. to study its unique characteristics.

The habitat of saprophytic oomycetes is primarily humid and wet soil. Recycling and rotting organic matter is one of the positive effects of saprophyte (Margulis et al., 2000; Kamoun et al., 2003). Oomycete plant pathogens are the source of many types of destructive diseases in crops. The genus of *Phytophthora* contained more than 60 species, most of which vigorously attack dicotyledon crops. Crops like potato, tomato, pepper, alfalfa, and soybeans were facing devastating problems by *Phytophthora* spp. (Agrios, 2005). *Phytophthora infestans* is the most critical disease-causing late blight of potatoes (Birch and Whisson, 2001; Kamoun, 2003). Worldwide, approximately US$6.7 billion annual loss occurs from the late blight of potatoes (Judelson, 2012). Many other economic-related diseases are caused by *Phytophthora* spp., such as *P. sojae* causing root rot disease in soybeans, *P. palmivora* and *Phytophthora megacarya* cause destructive disease in a black pod in cocoa, *P. cinnamomi* causes cranberry root rot and dieback of eucalypts, *P. ramorum* causes sudden death of the oak fish. *Albugo* and *Bremia* which cause white rust, mild mold caused by binding biotrophs *Plasmopara viticola* are not included in the *Phytophthora* genus. *Pythium* is another genus that is not included in *Phytophthora*, which comprises more than 100 species that cause many economic diseases.

### Phi Mediated Enhancement of Plant Resistance to Various Plant Pathogens

Phi has been documented as an effective pesticide to control several phytopathogenic organisms, such as nematode, fungi, and bacteria (Deliopoulos et al., 2010; Percival and Banks, 2014; Puerari et al., 2015). Phi induces a wide range of resistance to plant pathogens (Jost et al., 2015) and plays a vital role as the initial molecule of plant defense responses (Machinandiarena et al., 2012; Burra et al., 2014). In the occurrence of pathogenic bacteria, the use of 1.0 or 0.67 (*v*/*v*) Phi inhibits approximately 80% and 60% growth of *Streptomyces scabies* in potatoes, respectively (Lobato et al., 2010). When Phi is applied to the leaves of field-grown potato, the collected tubers showed less susceptibility to *Erwinia carotovora* inoculation, indicating that Phi induced systemic immune resistance (Lobato et al., 2011). In apples, the use of Phi highly decreased the prevalence of a blue mold caused by *Penicillium expansum* in injured and infected fruits (Amiri and Bompeix, 2011), while trunk injection was useful in the management of fire blight caused by *Erwinia amylovora* in apple trees (Aćimović et al., 2015). It was also used as a fungicide to control *Pseudoperonospora humuli*, which causes mold production in grapes (Salmon and Ware, 1925). In *Cucumis sativus*, Phi effectively contains *Pythium* spp. and suppresses disease by increasing the Phi concentration (Mofidnakhaei et al., 2016; Mofid Nakhaei et al., 2018). Researchers reported that Phi control *Plasmopara viticola*, but not *Oidium tuckeri* and *Pseudopezicula tracheiphila* in grape (Speiser et al., 2000). In their study, the use of Phi resulted in Phi residues in wine, which were nevertheless assessed toxic and safe. According to Reuveni et al. (2003), Phi treatment on apple fruits or trees gives a sufficient reduction of moldy core caused by *Alternaria alternate* and KPhi trunk injection in apple trees decreased *Erwinia amylovora* causal agent fire blight (Aćimović et al., 2015). In turnip, Chinese cabbage, and cabbage, the Phi treatment reduced disease severity of clubroot casual agent of *Plasmodiophora brassicae* (Abbasi and Lazarovits, 2006). In *Pinus* and *Pseudotsuga menziesii*, Cerqueira et al. (2017) reported that Phi prohibited *Fusarium circinatum* mycelium grown in a dose-dependent path. In corn, there is an efficient reduction in the occurrence and severity of *Peronosclero sporasorghi*, at the same time, yield increased by 73% (Panicker and Gangadharan, 1999). In *Glycine max* L. Silva et al. (2011) documented that the Phi decreased spot losses due to *Peronospora manshurica* incidence, while for the first time (Scott et al., 2015) witnessed an effective reduction of *Macrophomina phaseolina* by using a combination treatment with plant growth rhizobacteria (PGPR) grown under glasshouse conditions. Therefore, Phi can be a reasonable pesticide to prevent the incidence and prevalence of such pathogens in crops (Shafique et al., 2016). Because it is a global pathogen that causes many diseases including root rot, stem rot, and dry root stem rot around 500 plant species such as vegetables, fruits, and crops, for detailed information, see Table 4 (Khan, 2007).

**TABLE 4:** Plant diseases are suppressed by different sources of phosphite.

Plant	Disease	Causal agent	Experimental details	Phosphite source	References
Apple	Apple scab	*Venturia inaequalis*	Pot culture Bovine manure campus Vermiculite Sand	Potassium phosphite	Felipini et al. (2016)
Apple	Fire blight	*Erwinia amylovora*	Trunk injection (fiel d trial)	Potassium phosphite	Aćimović et al. (2015)
Apple	Blue mold	*Penicillium expansum*	*In vitro* and postharvest treatment	Potassium phosphite	Amiri and Bompeix (2011)
Apple	Moldy-Core	*Alternaria alternata*	Field trial	Potassium phosphite	Reuveni et al. (2003)
Bok Choy	Club rot	*Plasmodiophor abrassicae*	Field trial	AG3 Phosphonate	Abbasi and Lazarovits (2006)
Bristle oat	Nematode	*Meloidogyne marylandi*	Soil drenches	Phosphonic acid	Oka et al. (2007)
Cabbage	Club rot	*Plasmodiophor abrassicae*	Field trial	AG3 Phosphonate	Abbasi and Lazarovits (2006)
Corn	Foliar disease	*Phaeosphaeria maydis Cercosporazea emaydise*	Field trial	Potassium phosphite	Da Silva et al. (2020)
Corn	Root lesion nematode	*Exserohilum turcicum Pratylenchus brachyurus*	Pine bark, coconut fiber, and vermiculite	Manganese phosphite	Puerari et al. (2015)
Corn	Downy mildew	*Peronosclerospora sorghi*	Pot culture (peat, vermiculite, and soil)	Phosphonic acid	Panicker and Gangadharan (1999)
Cucumber	Downy mildew	*Pseudoperonos pora cubensis*	Pot culture Peat, perlite, and coco peat	Potassium phosphite	Ramezani et al. (2018)
Cucumber	Damping off	*Pythium ultimum* var.	Pot culture Peat, perlite, and coco peat	Potassiumphosphite	Mohammadi et al. (2020)
Grape	Downey mildew	*Plasmopara viticola*	Field trial	Potassium phosphite	Speiser et al. (2000)
Pecan	Pecan scab	*Fusicladium effusum*	Trunk injection	Potassium phosphite	Bock et al. (2013)
Pinus spp.	Pitch canker	*Fusarium circinatum*	Pot culture, peat, and perlite	Potassium phosphite	Cerqueira et al. (2017)
Potato	Tuber rot	*Rhizoctonia solani Fusarium solani Streptomyces scabies*	*In vitro* culture	Calcium phosphite Potassium phosphite Copper phosphite	Lobato et al. (2010)
Rice	Stem rot	*Nakataea oryzae* (Catt.)	Field trial	Potassium phosphite	Martinez-Medina et al. (2016)
Soybean	Charcoal root rot	*Macrophomina phaseolina*	*In vitro* assay	Manganese phosphite	Scott et al. (2015)
Soybean	White mold	*Sclerotinia sclerotiorum*	Pot culture Peat, pine bark, and vermiculite	Manganese phosphite	Novaes et al. (2019)
Wheat	Nematode	*Heterodera avenae*	Soil drenches	Phosphonic acid	Oka et al. (2007)

**FIGURE 6:**
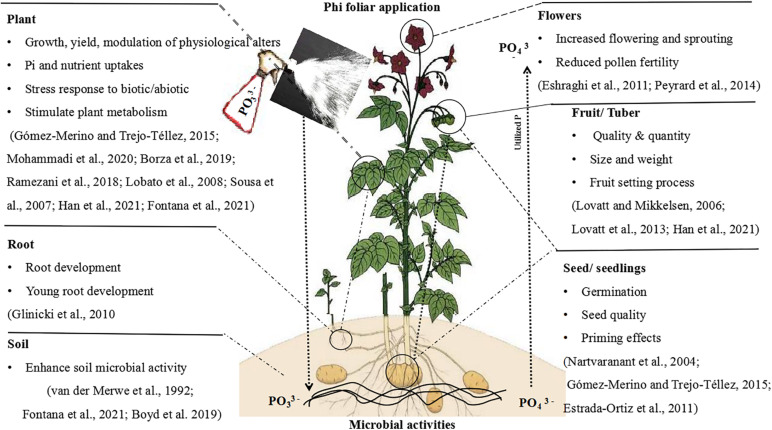
Schematic diagram with an example interpreting the main effects and physiological actions of Phi in the plant.

Plant-parasitic nematodes are highly economic important parasites. In maize, Phi application was useful for controlling *Pratylenchusbra chyurus* (Dias-Arieria et al., 2012). Phi’s capacity to accumulate plant defense response primed plants by phytoalexins synthesis (Dercks and Creasy, 1989). In addition, manganese Phi was effective against *Meloidogyne javanica* prevention in *Glycine max* and decreased the number of eggs/gram of root when applied 7 days prior to infection of nematodes in pest-resistant cultivar (Puerari et al., 2015). Similarly, Oka et al. (2007) found that the use of Phi in wheat and oats significantly suppressed *Heterodera avenae* and *Meloidogyne marylandi*, which stimulated Phi’s ability to confirm the synthesis of phytoalexins in plants (Dercks and Creasy, 1989; Han et al., 2021). Since nematodes are prevalent in some vegetables and crops, Phi is a highly effective bactericide to manage such pathogens in agriculture. The general response of plants to Phi application is shown in Figure 6).

### Phi-Mediated Gene Expression Modification Under Pathogen Stress

Previously, microarray analysis determined the changes in gene expression after Phi application and pathogen infection (Feldman et al., 2020). Their findings indicate a differential expression of 172 genes after Phi treatment and 22 Phi genes after pathogen infection, mainly the genes involved in signal transduction and defense responses, expressed (Feldman et al., 2020). Plant hormones play a central role in the expression of defense-related genes. Phi elevated the transcription of the genes involved in abscisic acid (ABA), ethylene biosynthesis, and mitogen-activated protein kinase cascade (MAPK) under *P. capsici* stress, which in turn elevated H_2_O_2_, stimulating expression and activities of antioxidant enzyme genes in pepper (*Capsicum annum* L.) (Liu et al., 2016). Also, the NAD kinase-2 mutation (NADK2) attenuates the disruption of ABA orifice closure and the ABA inhibition of light orifice opening. NADK2 disruption also disrupts ABA-stimulated H_2_O_2_ accumulation (Sun et al., 2018). Phi primed the expression of salicylic acid (SA) and pathogenesis-related protein transcripts, mobilized essential components of basal resistance, enhanced disease susceptibility, isolated phytoalexin deficiency, and negatively regulated MAPK in *Arabidopsis*, thus triggering SA-dependent defense responses following inoculation by *Hyaloperonospora arabidopsidis* (Massoud et al., 2012). Profiled proteins before and after Phi treatments in potato leaves demonstrated that priming plant leaves for 3 days induced the production of a large number of defense proteins against *P. infestans* (Lim et al., 2013).

In *Arabidopsis*, PR1, PR5, and NPR are marker genes involved in salicylic acid pathways, while THI2.1 and PDF1.2 are involved in jasmonic acid/ethylene pathways (Turner et al., 2002). Investigation includes the expression of the five defense-related genes at the transcription level by using quantitative real-time reverse transcription-polymerase chain reaction (qRT-PCR) (Eshraghi et al., 2011). Phi-treated *Arabidopsis* showed enhanced expression of these five genes *via* SA- or JA/ET-dependent pathways. In contrast, it was SA dependent rather than JA or ET dependent, and PR1 expression was induced, indicating a similarity to SAR with SA-dependent priming (Massoud et al., 2012).

## Phi-Mediated Enhancement of Plant Resistance to Abiotic Stresses

Phi also stimulates tolerance mechanisms against many abiotic stresses. In maize, the replacement of 1/4 Pi by Phi induced POD activity (Avila et al., 2011). Besides, Phi reduced the biomass stimulation of plants under low Pi supply, while no effect was observed in plants grown under efficient Pi supplement (Avila et al., 2011). In Phi-treated potato leaves exposed to ultraviolet stress, Phi increases photosynthetic pigments and psbA gene expression, which encodes the PS II reaction center protein D1 than control (Oyarburo et al., 2015). In addition, potatoes have been shown to prevent UV-B oxidative stress, thus mediating UV-B stress tolerance (Oyarburo et al., 2015). In our previous publication, we reported that Phi application significantly increased heat resistance in potato seedlings by evaluating morphological characteristics, photosynthetic apparatus, PS II efficiency, oxidative stress, and DNA damage level. Also, RNA sequencing was performed to investigate the role of Phi signals and mechanisms of basic heat resistance (Xi et al., 2020). The findings showed that Phi treatment is not only essential for better plant performance but also improves plant heating by reducing oxidative stress and DNA damage and improving the biological synthesis of osmolytes and defense metabolites in case of exposure to adverse thermal conditions. RNA-Seq showed that Phi’s immune responses to heat stress were regulated by reprogramming global gene expressions (Xi et al., 2020). Recently, it was noticed that the Phi application enhanced the PS pigments and proline accumulation in leaves under water-deficit stress with a value equal to those observed in irrigated control plants (Gonçalves et al., 2019). After the plant’s exposure to abiotic stress, Phi increases the large number of proteins involved in cell wall formation as tolerance inducers. Thus, Phi, as resistance or tolerance inducer is not limited only to trigger plant defense process against pathogens but also interact to abnormal condition and incuse abiotic stress resistance.

## Conclusion

Phi compounds in various plant species suppress most omycete pathogens. Currently, in agriculture systems, Phi is being used as a plant biostimulant to boost nutritional efficiency, yield, crop quality, production, and tolerance to biotic and abiotic stresses. Phi is also used as a highly useful resistance or defense inducer against several plant pathogens. Although, in nature, plants do not have the mechanisms to utilize Phi as a suitable P-source fertilizer. If not properly administered, its use can have devastating effects on plants. Because Phi appears as an effect of internal secretion, it may increase effective responses when used in low doses. However, when applied at high levels, adverse effects can result in cell damage or death. Therefore, its dosage and usage must be monitored to ensure a better response in a range of products. Here, we presented evidence that Phi can be used as biological stimulants in plant resistance. Phi as a plant biostimulant may activate several micro- and macromolecules and biochemical and physiological mechanisms that lead to the induction of plant tolerance response to biotic and abiotic stress factors and improve crop growth parameters and productivity. To confirm the efficiency of Phi application and prohibit adverse effects, the plant condition must first be considered. Besides, the details of testing and dosing of Phi for use must be timed appropriately to meet crop needs, which depends on crop genetic background, environmental signs and soil status, culture performance, chemical source, and Phi dosage. Using a new technology facilitated by science provides us this possibility to explore how and what extent Phi alters the molecular mechanism that triggers defense responses in several plant species. A piece of better knowledge on the molecular mechanisms of P utilization efficiency can be achieved. In crops, the proper application of Phi could allow plants to grow in soil with low Pi existence while addressing P degradation and herbicide resistance challenges.

As a result, Phi can stimulate positive effects on plants if appropriately combined with other protectant fungicides. Phi can act as biocellular stimulants in conventional cropping systems that increase crop yield, quality, and agronomic performance under stress conditions. Phi may also improve postharvest application on fruit quality. Also, it can be used to control pathogenic organisms, including bacteria, oomycete, fungi, and nematodes. Interestingly, Phi can be used in various methods, for example, as foliar spraying, trunk injection, postharvest treatment, hydroponic systems, and through fertilization, soil, and soil paints. However, the most influential method of application for different agricultural species and cultivars still needs to be studied. There are several other factors to consider regarding the global application of Phi in agriculture, including the development of pathogen resistance to Phi, the effect of Phi on soil microorganisms, its potential threat to public health, etc. Therefore, there is a need to study and document all these phenomena soon. Environmental impacts and new trends in international food markets regarding Phi’s residual level must be considered and ensured.

## Author Contributions

MM conceived the idea, wrote the manuscript, and drafted the figures and tables. YC and MA revised the manuscript. BJ and MW organized the tables. KY, TL, XH, LY, CD, BH, and SP organized the figures. GW-P conceived the idea and revised the manuscript. YQ conceived, organized, and financially supported the manuscript.

## Conflict of Interest

The authors declare that the research was conducted in the absence of any commercial or financial relationships that could be construed as a potential conflict of interest.

## Footnotes

1 www.frac.info

## References

[B1] Abbasi P. Lazarovits G. (2006). Effect of soil application of AG3 phosphonate on the severity of clubroot of bok choy and cabbage caused by *Plasmodiophora brassicae*. *Plant Dis.* 90 1517–1522. 10.1094/PD-90-1517 30780970

[B2] Aæimoviæ S. G. Zeng Q. Mcghee G. C. Sundin G. W. Wise J. C. (2015). Control of fire blight (*Erwinia amylovora*) on apple trees with trunk-injected plant resistance inducers and antibiotics and assessment of induction of pathogenesis-related protein genes. *Front. Plant Sci.* 6:16. 10.3389/fpls.2015.00016 25717330 PMC4323746

[B3] Adams D. J. (2004). Fungal cell wall chitinases and glucanases. *Microbiology* 150 2029–2035. 10.1099/mic.0.26980-0 15256547

[B4] Adhikari B. N. Hamilton J. P. Zerillo M. M. Tisserat N. Lévesque C. A. Buell C. R. (2013). Comparative genomics reveals insight into virulence strategies of plant pathogenic oomycetes. *PLoS One* 8:e0075072. 10.1371/journal.pone.0075072 24124466 PMC3790786

[B5] Agrios G. (2005). Plant diseases caused by fungi. *Plant Pathol.* 4. 10.1016/B978-0-08-047378-9.50017-8

[B6] Ahanger M. A. Agarwal R. (2017). Potassium up-regulates antioxidant metabolism and alleviates growth inhibition under water and osmotic stress in wheat (*Triticum aestivum* L). *Protoplasma* 254 1471–1486. 10.1007/s00709-016-1037-0 27783181

[B7] Alexandersson E. Mulugeta T. Lankinen Å Liljeroth E. Andreasson E. (2016). Plant resistance inducers against pathogens in solanaceae species—from molecular mechanisms to field application. *Int. J. Mol. Sci.* 17:1673. 10.3390/ijms17101673 27706100 PMC5085706

[B8] Ali M. B. Yu K.-W. Hahn E.-J. Paek K.-Y. (2006). Methyl jasmonate and salicylic acid elicitation induces ginsenosides accumulation, enzymatic and non-enzymatic antioxidant in suspension culture *Panax ginseng* roots in bioreactors. *Plant Cell Rep.* 25 613–620. 10.1007/s00299-005-0065-6 16463159

[B9] Amiri A. Bompeix G. (2011). Control of *Penicillium expansum* with potassium phosphite and heat treatment. *Crop Protect.* 30 222–227. 10.1016/j.cropro.2010.10.010

[B10] Arfaoui A. El Hadrami A. Adam L. R. Daayf F. (2020). Combining *Streptomyces hygroscopicus* and phosphite boosts soybean’s defense responses to *Phytophthora sojae*. *BioControl* 65 363–375. 10.1007/s10526-020-10000-7

[B11] Avila F. W. Faquin V. Araujo J. L. Marques D. J. Júnior P. M. R. Da Silva (2011). Phosphite supply affects phosphorus nutrition and biochemical responses in maize plants. *Aust. J. Crop Sci.* 5 646–645.

[B12] Ávila F. W. Faquin V. Da Silva, Lobato A. K. Ávila P. A. Marques D. J. (2013). Effect of phosphite supply in nutrient solution on yield, phosphorus nutrition and enzymatic behavior in common bean (’*Phaseolus vulgaris*’. L.) plants. *Aust. J. Crop Sci.* 7 713–722.

[B13] Belhaj R. (2017). *New Phytophthora Species in Western Australia: Pathogenicity and Control by Phosphite In Vitro and In Planta.* Perth: Murdoch University.

[B14] Banko T. Hong C. (2004). Evaluation of nutrient phosphite for the control of Phytophthora shoot blight on annual vinca. *J. Environ. Hortic.* 22 41–44. 10.24266/0738-2898-22.1.41

[B15] Barrett S. Shearer B. Hardy G. S. J. (2003). The efficacy of phosphite a pp lied after inoculation on the colonisation of *Banksia brownii* sterns by *Phytophthora cinnamomi*. *Australas. Plant Pathol.* 32 1–7. 10.1071/AP02061

[B16] Batista P. F. Müller C. Merchant A. Fuentes D. Silva-Filho R. D. O. Da Silva F. B. (2020). Biochemical and physiological impacts of zinc sulphate, potassium phosphite and hydrogen sulphide in mitigating stress conditions in soybean. *Physiol. Plant.* 168 456–472. 10.1111/ppl.13034 31600428

[B17] Bengtsson T. (2013). *Boosting Potato Defence Against Late Blight.* Alnarp: Acta Universitatis Agriculturae Sueciae.

[B18] Bengyella L. Iftikhar S. Nawaz K. Fonmboh D. J. Yekwa E. L. Jones R. C. (2019). Biotechnological application of endophytic filamentous bipolaris and curvularia: a review on bioeconomy impact. *World J. Microbiol. Biotechnol.* 35:69. 10.1007/s11274-019-2644-7 31011888

[B19] Biczak R. (2016). Quaternary ammonium salts with tetrafluoroborate anion: phytotoxicity and oxidative stress in terrestrial plants. *J. Hazard. Mater.* 304 173–185. 10.1016/j.jhazmat.2015.10.055 26551221

[B20] Birch P. R. Whisson S. C. (2001). Phytophthora infestans enters the genomics era. *Mol. Plant Pathol.* 2 257–263. 10.1046/j.1464-6722.2001.00073.x 20573013

[B21] Birch P. R. Bryan G. Fenton B. Gilroy E. M. Hein I. Jones J. T. (2012). Crops that feed the world 8: potato: are the trends of increased global production sustainable? *Food Security* 4 477–508. 10.1007/s12571-012-0220-1

[B22] Blum M. Gamper H. A. Waldner M. Sierotzki H. Gisi U. (2012). The cellulose synthase 3 (CesA3) gene of oomycetes: structure, phylogeny and influence on sensitivity to carboxylic acid amide (CAA) fungicides. *Fungal Biol.* 116 529–542. 10.1016/j.funbio.2012.02.003 22483051

[B23] Bock C. H. Brenneman T. B. Hotchkiss M. W. Wood B. W. (2013). Trunk applications of phosphite for the control of foliar and fruit scab on pecan. *Crop Prot.* 54 213–220.

[B24] Boller T. He S. Y. (2009). Innate immunity in plants: an arms race between pattern recognition receptors in plants and effectors in microbial pathogens. *Science* 324 742–744. 10.1126/science.1171647 19423812 PMC2729760

[B25] Borza T. Peters R. D. Gao X. Wang-Pruski G. (2019). Effects of phosphite on the in vitro growth of *Verticillium nonalfalfae* and *Verticillium dahliae* and on their in vivo ability to infect potato plants. *Eur. J. Plant Pathol.* 155 1333–1344. 10.1007/s10658-019-01859-z

[B26] Borza T. Peters R. Wu Y. Schofield A. Rand J. Ganga Z. (2017). Phosphite uptake and distribution in potato tubers following foliar and postharvest applications of phosphite-based fungicides for late blight control. *Ann. Appl. Biol.* 170 127–139. 10.1111/aab.12322

[B27] Borza T. Schofield A. Sakthivel G. Bergese J. Gao X. Rand J. (2014). Ion chromatography analysis of phosphite uptake and translocation by potato plants: dose-dependent uptake and inhibition of *Phytophthora infestans* development. *Crop Prot.* 56 74–81. 10.1016/j.cropro.2013.10.024

[B28] Burra D. D. Berkowitz O. Hedley P. E. Morris J. Resjö S. Levander F. (2014). Phosphite-induced changes of the transcriptome and secretome in Solanum tuberosum leading to resistance against *Phytophthora infestans*. *BMC Plant Biol.* 14:254. 10.1186/s12870-014-0254-y 25270759 PMC4192290

[B29] Candela M. Alcazar M. Espin A. Egea C. Almela L. (1995). Soluble phenolic acids in *Capsicum annuum* stems infected with *Phytophthora capsici*. *Plant Pathol.* 44 116–123. 10.1111/j.1365-3059.1995.tb02723.x

[B30] Carswell M. C. Grant B. R. Plaxton W. C. (1997). Disruption of the phosphate-starvation response of oilseed rape suspension cells by the fungicide phosphonate. *Planta* 203 67–74. 10.1007/s00050166 9299791

[B31] Cerqueira A. Alves A. Berenguer H. Correia B. Gómez-Cadenas A. Diez J. J. (2017). Phosphite shifts physiological and hormonal profile of *Monterey pine* and delays Fusarium circinatum progression. *Plant Physiol. Biochem.* 114 88–99. 10.1016/j.plaphy.2017.02.020 28284060

[B32] Chandrasekaran M. Belachew S. T. Yoon E. Chun S. C. (2017). Expression of β-1, 3-glucanase (GLU) and PAL ammonia-lyase (PAL) genes and their enzymes in tomato plants induced after treatment with *Bacillus subtilis* CBR05 against *Xanthomonas campestris* pv. vesicatoria. *J. Gen. Plant Pathol.* 83 7–13. 10.1007/s10327-016-0692-5

[B33] Coffey M. Joseph M. (1985). Effects of phosphorous acid and fosetyl-Al on the life cycle of *Phytophthora cinnamomi* and P. citricola. *Phytopathology* 75 1042–1046. 10.1094/Phyto-75-1042

[B34] Cohen Y. Coffey M. D. (1986). Systemic fungicides and the control of oomycetes. *Annu. Rev. Phytopathol.* 24 311–338. 10.1146/annurev.py.24.090186.001523

[B35] Constán-Aguilar C. Sánchez-Rodríguez E. Rubio-Wilhelmi M. Camacho M. Romero L. Ruiz J. (2014). Physiological and nutritional evaluation of the application of phosphite as a phosphorus source in cucumber plants. *Commun. Soil Sci. Plant Anal.* 45 204–222. 10.1080/00103624.2013.854374

[B36] Cui H. Tsuda K. Parker J. E. (2015). Effector-triggered immunity: from pathogen perception to robust defense. *Annu. Rev. Plant Biol.* 66 487–511. 10.1146/annurev-arplant-050213-040012 25494461

[B37] Daniel R. Guest D. (2005). Defence responses induced by potassium phosphonate in Phytophthora palmivora-challenged *Arabidopsis thaliana*. *Physiol. Mol. Plant Pathol.* 67 194–201. 10.1016/j.pmpp.2006.01.003

[B38] Danova-Alt R. Dijkema C. De Waard P. Koeck M. (2008). Transport and compartmentation of phosphite in higher plant cells–kinetic and 31P nuclear magnetic resonance studies. *Plant Cell Environ.* 31 1510–1521. 10.1111/j.1365-3040.2008.01861.x 18657056

[B39] Da Silva B. C. Couto O. D. Obata H. T. De Lima M. M. Bonani F. D. De Oliveira C. E. (2020). Optical absorption exhibits pseudo-direct band gap of wurtzite gallium phosphide. *Sci. Rep.* 10 1–7. 32404930 10.1038/s41598-020-64809-4 PMC7221080

[B40] Debnath B. Irshad M. Mitra S. Li M. Rizwan H. M. Liu S. (2018). Acid rain deposition modulates photosynthesis, enzymatic and non-enzymatic antioxidant activities in tomato. *Int. J. Environ. Res.* 12 203–214. 10.1007/s41742-018-0084-0

[B41] Deliopoulos T. Kettlewell P. S. Hare M. C. (2010). Fungal disease suppression by inorganic salts: a review. *Crop Prot.* 29 1059–1075. 10.1016/j.cropro.2010.05.011

[B42] Dercks W. Creasy L. (1989). Influence of fosetyl-Al on phytoalexin accumulation in the Plasmopara viticola-grapevine interaction. *Physiol. Mol. Plant Pathol.* 34 203–213. 10.1016/0885-5765(89)90044-1

[B43] Dias M. Figueiredo P. Duarte I. Gil A. Santos C. (2014). Different responses of young and expanded lettuce leaves to fungicide Mancozeb: chlorophyll fluorescence, lipid peroxidation, pigments and proline content. *Photosynthetica* 52 148–151. 10.1007/s11099-014-0016-y

[B44] Dias-Arieria C. R. Marini P. M. Fontana L. F. Roldi M. Da Silva T. R. B. (2012). Effect of Azospirillum Brasilense, stimulate^®^ and Potassium Phosphite to control Pratylenchus Brachyurus in Soybean and Maize [Efeito De Azospirillum Brasilense, Stimulate^®^ E Fosfito De Potássio No Controle De Pratylenchus Brachyurus Em Soja E Milho]. *Nematropica* 42 170–175.

[B45] Djébali N. Mhadhbi H. Lafitte C. Dumas B. Esquerré-Tugayé M.-T. Aouani M. E. (2011). Hydrogen peroxide scavenging mechanisms are components of *Medicago truncatula* partial resistance to *Aphanomyces euteiches*. *Eur. J. Plant Pathol.* 131 559. 10.1007/s10658-011-9831-1

[B46] El-Hamalawi Z. Menge J. Adams C. (1995). Methods of fosetyl-Al application and phosphonate levels in avocado tissue needed to control stem canker caused by Phytophthora citricola. *Plant Dis.* 79 770–778. 10.1094/PD-79-0770

[B47] Eshraghi L. Anderson J. P. Aryamanesh N. Mccomb J. A. Shearer B. Hardy G. S. J. (2014). Suppression of the auxin response pathway enhances susceptibility to *Phytophthora cinnamomi* while phosphite-mediated resistance stimulates the auxin signalling pathway. *BMC Plant Biol.* 14:68. 10.1186/1471-2229-14-68 24649892 PMC3999932

[B48] Eshraghi L. Anderson J. Aryamanesh N. Shearer B. Mccomb J. Hardy G. S. (2011). Phosphite primed defence responses and enhanced expression of defence genes in *Arabidopsis thaliana* infected with *Phytophthora cinnamomi*. *Plant Pathol.* 60 1086–1095. 10.1111/j.1365-3059.2011.02471.x

[B49] Estrada-Ortiz E. Trejo-Téllez L. Gómez-Merino F. Núñez-Escobar R. Sandoval-Villa M. (2011). “Phosphite on growth and fruit quality in strawberry,” in *Proceedings of the II International Symposium on Soilless Culture and Hydroponics 947*, (Belgium: ISHS). 10.17660/ActaHortic.2012.947.35

[B50] Fagundes-Nacarath I. Debona D. Oliveira A. Hawerroth C. Rodrigues F. (2018). Biochemical responses of common bean to white mold potentiated by phosphites. *Plant Physiol. Biochem.* 132 308–319. 10.1016/j.plaphy.2018.09.016 30248517

[B51] Fairbanks M. Hardy G. S. J. Mccomb J. (2001). The effect of phosphite on the sexual reproduction of some annual species of the jarrah (*Eucalyptus marginata*) forest of southwest Western Australia. *Sexual Plant Reprod.* 13 315–321. 10.1007/s004970100072

[B52] Feldman M. L. Guzzo M. C. Machinandiarena M. F. Rey-Burusco M. F. Beligni M. V. Di Rienzo J. (2020). New insights into the molecular basis of induced resistance triggered by potassium phosphite in potato. *Physiol. Mol. Plant Pathol.* 109:101452. 10.1016/j.pmpp.2019.101452

[B53] Felipini R. B. Boneti J. I. Katsurayama Y. Neto A. C. R. Veleirinho B. Maraschin M. (2016). Apple scab control and activation of plant defence responses using potassium phosphite and chitosan. *Eur. J. Plant Pathol.* 145 929–939. 10.1007/s10658-016-0881-2

[B54] Fernandes L. H. M. De Oliveira Silveira H. R. De Souza K. R. D. De Resende M. L. V. Alves J. D. (2014). Inductors of resistance and their role in photosynthesis and antioxidant system activity of coffee seedlings. *Am. J. Plant Sci.* 5 3710–3716. 10.4236/ajps.2014.525387

[B55] Fo H. Oudemans P. Coffey M. D. (1990). Mitochondrial and nuclear DNA diversity within six species ofPhytophthora. *Exp. Mycol.* 14 18–31. 10.1016/0147-5975(90)90083-6

[B56] Fontana M. Bragazza L. Guillaume T. Santonja M. Buttler A. Elfouki S. (2021). Valorization of calcium phosphite waste as phosphorus fertilizer: effects on green manure productivity and soil properties. *J. Environ. Manag.* 285:112061. 10.1016/j.jenvman.2021.112061 33582477

[B57] Forouzanfar M. H. Afshin A. Alexander L. T. Anderson H. R. Bhutta Z. A. Biryukov S. (2016). Global, regional, and national comparative risk assessment of 79 behavioural, environmental and occupational, and metabolic risks or clusters of risks, 1990–2015: a systematic analysis for the Global Burden of Disease Study 2015. *Lancet* 388 1659–1724. 10.1016/S0140-6736(16)31679-8 27733284 PMC5388856

[B58] Förster H. Adaskaveg J. E. Kim D. H. Stanghellini M. E. (1998). Effect of phosphite on tomato and pepper plants and on susceptibility of pepper to Phytophthora root and crown rot in hydroponic culture. *Plant Dis.* 82 1165–1170. 10.1094/PDIS.1998.82.10.1165 30856781

[B59] Gent D. H. Block M. Claassen B. J. (2020). High levels of insensitivity to phosphonate fungicides in *Pseudoperonospora humuli*. *Plant Dis.* 104 1400–1406. 10.1094/PDIS-10-19-2067-RE 32196418

[B60] Gilbert G. A. Knight J. D. Vance C. P. Allan D. L. (2000). Proteoid root development of phosphorus deficient lupin is mimicked by auxin and phosphonate. *Ann. Bot.* 85 921–928. 10.1006/anbo.2000.1133

[B61] Gimenez-Ibanez S. Zamarreño A. M. García-Mina J. M. Solano R. (2019). An evolutionarily ancient immune system governs the interactions between *Pseudomonas syringae* and an early-diverging land plant lineage. *Curr. Biol.* 29 2270–2281. e2274. 10.1016/j.cub.2019.05.079 31303486

[B62] Glinicki R. Sas-Paszt L. Jadczuk-Tobjasz E. (2010). The effect of plant stimulant/fertilizer “resistim” on growth and development of strawberry plants. *J. Fruit Ornamental Plant Res.* 18 111–124.

[B63] Gómez-Merino F. C. Trejo-Téllez L. I. (2015). Biostimulant activity of phosphite in horticulture. *Sci. Hortic.* 196 82–90. 10.1016/j.scienta.2015.09.035

[B64] Gonçalves K. S. Da Silva, Paz V. P. De Lima Silva F. Hongyu K. De Almeida W. F. (2019). Potassium phosphite and water deficit: physiological response of eucalyptus using multivariate analysis. *J. Agric. Sci.* 11. 10.5539/jas.v11n3p565

[B65] Govers F. Gijzen M. (2006). Phytophthora genomics: the plant destroyers’ genome decoded. *Mol. Plant Microbe Interact.* 19 1295–1301. 10.1094/MPMI-19-1295 17153913

[B66] Greenwood N. (1984). *Oxoacids of Phosphorus and Their Salts.* Oxford: Pergamon Press.

[B67] Greenwood N. N. Earnshaw A. (1997). *Chemistry of the Elements.* Italy: Piccin Nuova Libraria-SpA Padova.

[B68] Greenwood N. N. Earnshaw A. (2012). *Chemistry of the Elements.* Amsterdam: Elsevier.

[B69] Griffith J. M. Coffey M. D. Grant B. R. (1993). Phosphonate inhibition as a function of phosphate concentration in isolates of *Phytophthora palmivora*. *Microbiology* 139 2109–2116. 10.1099/00221287-139-9-2109

[B70] Groves E. Howard K. Hardy G. Burgess T. (2015). Role of salicylic acid in phosphite-induced protection against Oomycetes; a *Phytophthora cinnamomi-Lupinus augustifolius* model system. *Eur. J. Plant Pathol.* 141 559–569. 10.1007/s10658-014-0562-y

[B71] Guest D. I. Pegg K. G. Whiley A. W. (1995). Control of Phytophthora diseases of tree crops using trunk-injected phosphonates. *Hortic. Rev.* 17 299–330. 10.1002/9780470650585.ch9

[B72] Guest D. Grant B. (1991). The complex action of phosphonates as antifungal agents. *Biol. Rev.* 66 159–187. 10.1111/j.1469-185X.1991.tb01139.x

[B73] Guthrie J. P. (1979). Tautomerization equilibria for phosphorous acid and its ethyl esters, free energies of formation of phosphorous and phosphonic acids and their ethyl esters, and p K a values for ionization of the P—H bond in phosphonic acid and phosphonic esters. *Can. J. Chem.* 57 236–239. 10.1139/v79-039 33356898

[B74] Han X. Xi Y. Zhang Z. Mohammadi M. A. Joshi J. Borza T. (2021). Effects of phosphite as a plant biostimulant on metabolism and stress response for better plant performance in Solanum tuberosum. *Ecotoxicol. Environ. Saf.* 210:111873. 10.1016/j.ecoenv.2020.111873 33418157

[B75] Hardham A. R. (2001). The cell biology behind Phytophthora pathogenicity. *Australas. Plant Pathol.* 30 91–98. 10.1071/AP01006

[B76] Hardham A. R. (2007). Cell biology of plant–oomycete interactions. *Cell Microbiol.* 9 31–39. 10.1111/j.1462-5822.2006.00833.x 17081190

[B77] Hardham A. R. Blackman L. M. (2018). *Phytophthora cinnamomi*. *Mol. Plant Pathol.* 19 260–285. 10.1111/mpp.12568 28519717 PMC6637996

[B78] Hardy G. S. J. Barrett S. Shearer B. (2001). The future of phosphite as a fungicide to control the soilborne plant pathogen *Phytophthora cinnamomi* in natural ecosystems. *Australas. Plant Pathol.* 30 133–139. 10.1071/AP01012

[B79] Hasanuzzaman M. Bhuyan M. Anee T. I. Parvin K. Nahar K. Mahmud J. A. (2019). Regulation of ascorbate-glutathione pathway in mitigating oxidative damage in plants under abiotic stress. *Antioxidants* 8:384. 10.3390/antiox8090384 31505852 PMC6770940

[B80] Hasanuzzaman M. Bhuyan M. Mahmud J. Nahar K. Mohsin S. Parvin K. (2018). Interaction of sulfur with phytohormones and signaling molecules in conferring abiotic stress tolerance to plants. *Plant Signal. Behav.* 13:e1477905. 10.1080/15592324.2018.1477905 29939817 PMC6103289

[B81] Heinonen J. K. (2001). *Biological Role of Inorganic Pyrophosphate.* Berlin: Springer. 10.1007/978-1-4615-1433-6

[B82] Jackson A. W. Andrews G. A. (2000). *Turning Points 2000: Educating Adolescents in the 21st Century.* New York, NY: Teachers College Press.

[B83] Jackson T. Burgess T. Colquhoun I. Hardy G. S. (2000). Action of the fungicide phosphite on *Eucalyptus marginata* inoculated with *Phytophthora cinnamomi*. *Plant Pathol.* 49 147–154. 10.1046/j.1365-3059.2000.00422.x

[B84] Jiang F. Hartung W. (2008). Long-distance signalling of abscisic acid (ABA): the factors regulating the intensity of the ABA signal. *J. Exp. Bot.* 59 37–43. 10.1093/jxb/erm127 17595196

[B85] Jiang S. Han S. He D. Cao G. Fang K. Xiao X. (2019). The accumulation of phenolic compounds and increased activities of related enzymes contribute to early defense against walnut blight. *Physiol. Mol. Plant Pathol.* 108:101433. 10.1016/j.pmpp.2019.101433

[B86] Johnson D. A. Inglis D. A. Miller J. S. (2004). Control of potato tuber rots caused by oomycetes with foliar applications of phosphorous acid. *Plant Dis.* 88 1153–1159. 10.1094/PDIS.2004.88.10.1153 30795259

[B87] Jones J. D. Dangl J. L. (2006). The plant immune system. *Nature* 444 323–329. 10.1038/nature05286 17108957

[B88] Jost R. Pharmawati M. Lapis-Gaza H. R. Rossig C. Berkowitz O. Lambers H. (2015). Differentiating phosphate-dependent and phosphate-independent systemic phosphate-starvation response networks in *Arabidopsis thaliana* through the application of phosphite. *J. Exp. Bot.* 66 2501–2514. 10.1093/jxb/erv025 25697796 PMC4986860

[B89] Judelson H. S. (2012). Dynamics and innovations within oomycete genomes: insights into biology, pathology, and evolution. *Eukaryot. Cell* 11 1304–1312. 10.1128/EC.00155-12 22923046 PMC3486027

[B90] Jung S. Kim J. S. Cho K. Y. Tae G. S. Kang B. G. (2000). Antioxidant responses of cucumber (*Cucumis sativus*) to photoinhibition and oxidative stress induced by norflurazon under high and low PPFDs. *Plant Sci.* 153 145–154. 10.1016/S0168-9452(99)00259-9 10717320

[B91] Kamoun P. Belardinelli M. C. Chabli A. Lallouchi K. Chadefaux-Vekemans B. (2003). Endogenous hydrogen sulfide overproduction in Down syndrome. *Am. J. Med. Genet. Part A* 116 310–311. 10.1002/ajmg.a.10847 12503113

[B92] Kamoun S. (2003). Molecular genetics of pathogenic oomycetes. *Eukaryot. Cell* 2 191–199. 10.1128/EC.2.2.191-199.2003 12684368 PMC154851

[B93] Kamoun S. (2006). A catalogue of the effector secretome of plant pathogenic oomycetes. *Annu. Rev. Phytopathol.* 44 41–60. 10.1146/annurev.phyto.44.070505.143436 16448329

[B94] Kapoor D. Singh S. Kumar V. Romero R. Prasad R. Singh J. (2019). Antioxidant enzymes regulation in plants in reference to reactive oxygen species (ROS) and reactive nitrogen species (RNS). *Plant Gene* 19:100182. 10.1016/j.plgene.2019.100182

[B95] Khan S. N. (2007). Macrophomina phaseolina as causal agent for charcoal rot of sunflower. *Mycopath* 5 111–118.

[B96] King M. Reeve W. Van Der Hoek M. B. Williams N. Mccomb J. O’brien P. A. (2010). Defining the phosphite-regulated transcriptome of the plant pathogen *Phytophthora cinnamomi*. *Mol. Genet. Genom.* 284 425–435. 10.1007/s00438-010-0579-7 20882389

[B97] Kraszewski A. Stawinski J. (2007). H-Phosphonates: versatile synthetic precursors to biologically active phosphorus compounds. *Pure Appl. Chem.* 79 2217–2227. 10.1351/pac200779122217

[B98] Lamour K. H. Win J. Kamoun S. (2007). Oomycete genomics: new insights and future directions. *FEMS Microbiol. Lett.* 274 1–8. 10.1111/j.1574-6968.2007.00786.x 17559387

[B99] Landschoot P. Cook J. (2005). *Understanding the Phosphonates Products. Department of Crop and Soil Sciences.* University Park, PA: The Pennsylvania State University.

[B100] Latijnhouwers M. De Wit P. J. Govers F. (2003). Oomycetes and fungi: similar weaponry to attack plants. *Trends Microbiol.* 11 462–469. 10.1016/j.tim.2003.08.002 14557029

[B101] Laxa M. Liebthal M. Telman W. Chibani K. Dietz K.-J. (2019). The role of the plant antioxidant system in drought tolerance. *Antioxidants* 8:94. 10.3390/antiox8040094 30965652 PMC6523806

[B102] Liljeroth E. Lankinen Å Wiik L. Burra D. D. Alexandersson E. Andreasson E. (2016). Potassium phosphite combined with reduced doses of fungicides provides efficient protection against potato late blight in large-scale field trials. *Crop Prot.* 86 42–55. 10.1016/j.cropro.2016.04.003

[B103] Lim S. (2012). *Analysis of Changes in the Potato Leaf Proteome Triggered by Phosphite Reveals Functions Associated with Induced Resistance Against Phytophthora infestans.* Canada: Dalhouse University.

[B104] Lim S. Borza T. Peters R. D. Coffin R. H. Al-Mughrabi K. I. Pinto D. M. (2013). Proteomics analysis suggests broad functional changes in potato leaves triggered by phosphites and a complex indirect mode of action against *Phytophthora infestans*. *J. Proteomics* 93 207–223. 10.1016/j.jprot.2013.03.010 23542353

[B105] Liu J. Sun Z. Zou Y. Li W. He F. Huang X. (2020). Pre-and postharvest measures used to control decay and mycotoxigenic fungi in potato (*Solanum tuberosum* L.) during storage. *Critical Rev. Food Sci. Nutr.* 4 1–14. 10.1080/10408398.2020.1818688 32924541

[B106] Liu P. Li B. Lin M. Chen G. Ding X. Weng Q. (2016). Phosphite-induced reactive oxygen species production and ethylene and ABA biosynthesis, mediate the control of *Phytophthora capsici* in pepper (*Capsicum annuum*). *Funct. Plant Biol.* 43 563–574. 10.1071/FP16006 32480486

[B107] Lobato M. C. Daleo G. R. Andreu A. B. Olivieri F. P. (2017). Cell wall reinforcement in the potato tuber periderm after crop treatment with potassium phosphite. *Potato Res.* 61 19–29. 10.1007/s11540-017-9349-9

[B108] Lobato M. C. Machinandiarena M. F. Tambascio C. Dosio G. A. Caldiz D. O. Daleo G. R. (2011). Effect of foliar applications of phosphite on post-harvest potato tubers. *Eur. J. Plant Pathol.* 130 155–163. 10.1007/s10658-011-9741-2

[B109] Lobato M. Olivieri F. Altamiranda E. G. Wolski E. Daleo G. Caldiz D. (2008). Phosphite compounds reduce disease severity in potato seed tubers and foliage. *Eur. J. Plant Pathol.* 122 349–358. 10.1007/s10658-008-9299-9

[B110] Lobato M. Olivieri F. Daleo G. Andreu A. (2010). Antimicrobial activity of phosphites against different potato pathogens. *J. Plant Dis. Prot.* 117 102–109. 10.1007/BF03356343

[B111] Lombardi L. Sebastiani L. (2005). Copper toxicity in *Prunus cerasifera*: growth and antioxidant enzymes responses of in vitro grown plants. *Plant Sci.* 168 797–802. 10.1016/j.plantsci.2004.10.012

[B112] Lovatt C. Mikkelsen R. (2006). Phosphite fertilizers: What are they? Can you use them? What can they do. *Better Crops* 90 11–13.

[B113] Luis A. Corpas F. J. López-Huertas E. Palma J. M. (2018). “Plant superoxide dismutases: function under abiotic stress conditions,” in *Antioxidants and Antioxidant Enzymes in Higher Plants*, eds Gupta D. K. Palma J. M. Corpas F. J. (Berlin: Springer).

[B114] Machinandiarena M. F. Lobato M. C. Feldman M. L. Daleo G. R. Andreu A. B. (2012). Potassium phosphite primes defense responses in potato against *Phytophthora infestans*. *J. Plant Physiol.* 169 1417–1424. 10.1016/j.jplph.2012.05.005 22727804

[B115] Mailloux R. J. (2016). Application of mitochondria-targeted pharmaceuticals for the treatment of heart disease. *Curr. Pharm. Des.* 22 4763–4779. 10.2174/1381612822666160629070914 27356774

[B116] Mao Y. Tyler B. M. (1996). Cloning and sequence analysis of elicitin genes of *Phytophthora sojae*. *Fungal Genet. Biol.* 20 169–172. 10.1006/fgbi.1996.0031 8810521

[B117] Margulis L. Dolan M. F. Guerrero R. (2000). The chimeric eukaryote: origin of the nucleus from the karyomastigont in amitochondriate protists. *Proc. Natl. Acad. Sci. U.S.A.* 97 6954–6959. 10.1073/pnas.97.13.6954 10860956 PMC34369

[B118] Martinez-Medina A. Flors V. Heil M. Mauch-Mani B. Pieterse C. M. Pozo M. J. (2016). Recognizing plant defense priming. *Trends Plant Sci.* 21 818–822. 10.1016/j.tplants.2016.07.009 27507609

[B119] Martins I. M. Meirinho S. Costa R. Cravador A. Choupina A. (2019). Cloning, characterization, in vitro and in planta expression of a necrosis-inducing Phytophthora protein 1 gene npp1 from *Phytophthora cinnamomi*. *Mol. Biol. Rep.* 46 6453–6462. 10.1007/s11033-019-05091-0 31571106

[B120] Massoud K. Barchietto T. Le Rudulier T. Pallandre L. Didierlaurent L. Garmier M. (2012). Dissecting phosphite-induced priming in Arabidopsis infected with *Hyaloperonospora arabidopsidis*. *Plant Physiol.* 159 286–298. 10.1104/pp.112.194647 22408091 PMC3375965

[B121] Mayton H. Myers K. Fry W. (2008). Potato late blight in tubers—The role of foliar phosphonate applications in suppressing pre-harvest tuber infections. *Crop Prot.* 27 943–950. 10.1016/j.cropro.2007.11.014

[B122] McCarren K. Mccomb J. Shearer B. Hardy G. S. J. (2005). The role of chlamydospores of *Phytophthora cinnamomi*—a review. *Australas. Plant Pathol.* 34 333–338. 10.1071/AP05038

[B123] McDonald A. E. Grant B. R. Plaxton W. C. (2001). Phosphite (phosphorous acid): its relevance in the environment and agriculture and influence on plant phosphate starvation response. *J. Plant Nutr.* 24 1505–1519. 10.1081/PLN-100106017

[B124] Mcleod A. Masikane S. L. Novela P. Ma J. Mohale P. Nyoni M. (2018). Quantification of root phosphite concentrations for evaluating the potential of foliar phosphonate sprays for the management of avocado root rot. *Crop Prot.* 103 87–97. 10.1016/j.cropro.2017.09.013

[B125] Miller J. S. Olsen N. Woodell L. Porter L. D. Clayson S. (2006). Post-harvest applications of zoxamide and phosphite for control of potato tuber rots caused by oomycetes at harvest. *Am. J. Potato Res.* 83 269–278. 10.1007/BF02872163

[B126] Mofid Nakhaei M. Abdossi V. Dehestani A. Pirdashti H. Babaeizad V. (2018). Enhanced defense responses in *Pythium ultimum*-challenged cucumber plants induced by potassium phosphite. *J. Plant Mol. Breed.* 6 24–33.

[B127] Mofidnakhaei M. Abdossi V. Dehestani A. Pirdashti H. Babaeizad V. (2016). Potassium phosphite affects growth, antioxidant enzymes activity and alleviates disease damage in cucumber plants inoculated with *Pythium ultimum*. *Arch. Phytopathol. Plant Prot.* 49 207–221. 10.1080/03235408.2016.1180924

[B128] Mohammadi M. A. Han X. Zhang Z. Xi Y. Boorboori M. Wang-Pruski G. (2020). Phosphite application alleviates *Pythophthora infestans* by modulation of photosynthetic and physio-biochemical metabolites in potato leaves. *Pathogens* 9:170. 10.3390/pathogens9030170 32121090 PMC7157663

[B129] Mohammadi M. Zhang Z. Xi Y. Han H. Lan F. Zhang B. (2019). Effects of Potassium Phosphite on biochemical contents and enzymatic activities of Chinese potatoes inoculated by *Phytophthora infestans*. *Appl. Ecol. Environ. Res.* 17 4499–4514. 10.15666/aeer/1702_44994514

[B130] Nartvaranant P. Hamill S. Leonardi J. Whiley A. Subhadrabandhu S. (2004). Seasonal effects of foliar application of phosphonate on phosphonate translocation, in vitro pollen viability and pollen germination inHass’ avocado (Persea americana Mill.). *J. Hortic. Sci. Biotechnol.* 79 91–96. 10.1080/14620316.2004.11511741

[B131] Nath M. Bhatt D. Bhatt M. D. Prasad R. Tuteja N. (2018). “Microbe-mediated enhancement of nitrogen and phosphorus content for crop improvement,” in *Crop Improvement Through Microbial Biotechnology*, eds Prasad R. Gill S. S. Tuteja N. (Amsterdam: Elsevier). 10.1016/B978-0-444-63987-5.00014-1

[B132] Nejat N. Mantri N. (2017). Plant immune system: crosstalk between responses to biotic and abiotic stresses the missing link in understanding plant defence. *Curr. Issues Mol. Biol.* 23 1–16. 10.21775/cimb.023.001 28154243

[B133] Nelson D. L. Cox M. M. Lehninger A. L. (2008). *Principles of Biochemistry.* New York: W. H. Freeman.

[B134] Norton G. W. Swinton S. M. (2018). “Precision agriculture: global prospects and environmental implications,” in *Proceedings of the Twenty-fouth International Conference of Agricultural Economists: Incentives, Institutions, Infrastructure and Innovations*, (Milton Park: Routledge).

[B135] Nouet C. Motte P. Hanikenne M. (2011). Chloroplastic and mitochondrial metal homeostasis. *Trends Plant Sci.* 16 395–404. 10.1016/j.tplants.2011.03.005 21489854

[B136] Novaes M. Debona D. Fagundes-Nacarath I. Brás V. Rodrigues F. (2019). Physiological and biochemical responses of soybean to white mold affected by manganese phosphite and fluazinam. *Acta Physiol. Plant.* 41:186. 10.1007/s11738-019-2976-9

[B137] Oka Y. Tkachi N. Mor M. (2007). Phosphite inhibits development of the nematodes *Heterodera avenae* and Meloidogyne marylandi in cereals. *Phytopathology* 97 396–404. 10.1094/PHYTO-97-4-0396 18943279

[B138] Olivieri F. P. Feldman M. L. Machinandiarena M. F. Lobato M. C. Caldiz D. O. Daleo G. R. (2012). Phosphite applications induce molecular modifications in potato tuber periderm and cortex that enhance resistance to pathogens. *Crop Prot.* 32 1–6. 10.1016/j.cropro.2011.08.025

[B139] Orbović V. Syvertsen J. P. Bright D. Van Clief D. L. Graham J. H. (2008). Citrus seedling growth and susceptibility to root rot as affected by phosphite and phosphate. *J. Plant Nutr.* 31 774–787. 10.1094/PDIS.1998.82.6.683 30857022

[B140] Oren Y. Yogev E. (2002). Acquired resistance to Phytophthora root rot and brown rot in citrus seedlings induced by potassium phosphite/Durch Kaliumphosphit induzierte erworbene Resistenz gegen Phytophthora-, Wurzel-und Braunfäule an Zitrus-Jungpflanzen. *J. Plant Dis. Prot.* 109 279–285.

[B141] Oyarburo N. S. Machinandiarena M. F. Feldman M. L. Daleo G. R. Andreu A. B. Olivieri F. P. (2015). Potassium phosphite increases tolerance to UV-B in potato. *Plant Physiol. Biochem.* 88 1–8. 10.1016/j.plaphy.2015.01.003 25596554

[B142] Panicker S. Gangadharan K. (1999). Controlling downy mildew of maize caused by *Peronosclerospora sorghi* by foliar sprays of phosphonic acid compounds. *Crop Prot.* 18 115–118. 10.1016/S0261-2194(98)00101-X

[B143] Peluffo L. Lia V. Troglia C. Maringolo C. Norma P. Escande A. (2010). Metabolic profiles of sunflower genotypes with contrasting response to *Sclerotinia sclerotiorum* infection. *Phytochemistry* 71 70–80. 10.1016/j.phytochem.2009.09.018 19853265

[B144] Percival G. C. Banks J. M. (2014). Evaluation of plant defence activators for the potential control of Pseudomonas syringae pv. aesculi. *Arboric. J.* 36 76–88. 10.1080/03071375.2014.921396

[B145] Peyrard S. Deckers T. De Maeyer L. Sirven C. Latorse M.-P. (2015). “Involvement of a plant defenses enhancer Fosetyl-Al in flowering induction: from the field to the genes,” in *Proceedings of the XII International Pear Symposium 1094*, 83–89.

[B146] Puerari H. H. Dias-Arieira C. R. Cardoso M. R. Hernandes I. Brito O. D. C. (2015). Resistance inducers in the control of root lesion nematodes in resistant and susceptible cultivars of maize. *Phytoparasitica* 43 383–389. 10.1007/s12600-014-0447-9

[B147] Raggi V. (1978). The CO_2_ compensation point, photosynthesis and respiration in rust infected bean leaves. *Physiol. Plant Pathol.* 13 135–139. 10.1016/0048-4059(78)90080-2

[B148] Ramezani M. Rahmani F. Dehestani A. (2017a). Study of physio-biochemical responses elicited by potassium phosphite in downy mildew-infected cucumber plants. *Arch. Phytopathol. Plant Prot.* 50 540–554. 10.1080/03235408.2017.1341140

[B149] Ramezani M. Rahmani F. Dehestani A. (2017b). The effect of potassium phosphite on PR genes expression and the phenylpropanoid pathway in cucumber (*Cucumis sativus*) plants inoculated with *Pseudoperonospora cubensis*. *Sci. Hortic.* 225 366–372. 10.1016/j.scienta.2017.07.022

[B150] Ramezani M. Ramezani F. Rahmani F. Dehestani A. (2018). Exogenous potassium phosphite application improved PR-protein expression and associated physio-biochemical events in cucumber challenged by *Pseudoperonospora cubensis*. *Sci. Hortic.* 234 335–343. 10.1016/j.scienta.2018.02.042

[B151] Rebollar-Alviter A. Madden L. Ellis M. (2007). Pre-and post-infection activity of azoxystrobin, pyraclostrobin, mefenoxam, and phosphite against leather rot of strawberry, caused by *Phytophthora cactorum*. *Plant Dis.* 91 559–564. 10.1094/PDIS-91-5-0559 30780701

[B152] Reuveni M. Sheglov D. Cohen Y. (2003). Control of moldy-core decay in apple fruits by β-aminobutyric acids and potassium phosphites. *Plant Dis.* 87 933–936. 10.1094/PDIS.2003.87.8.933 30812798

[B153] Rocafort M. Fudal I. Mesarich C. H. (2020). Apoplastic effector proteins of plant-associated fungi and oomycetes. *Curr. Opin. Plant Biol.* 56 9–19. 10.1016/j.pbi.2020.02.004 32247857

[B154] Roháček K. Soukupová J. Barták M. (2008). Chlorophyll fluorescence: a wonderful tool to study plant physiology and plant stress. *Plant Cell Compartments Selected Top.* 2008 41–104.

[B155] Saed-Moucheshi A. Shekoofa A. Pessarakli M. (2014). Reactive oxygen species (ROS) generation and detoxifying in plants. *J. Plant Nutr.* 37 1573–1585. 10.1080/01904167.2013.868483

[B156] Saindrenan P. Barchietto T. Bompeix G. (1990). Effects of phosphonate on the elicitor activity of culture filtrates of P*hytophthora cryptogea* in *Vigna unguiculata*. *Plant Sci.* 67 245–251. 10.1016/0168-9452(90)90249-N

[B157] Salmon E. Ware W. (1925). On the presence of a perennial mycelium in *Pseudoperonospora humuli* (Miyabe & Takah.) Wils. *Nature* 116 134–135. 10.1038/116134b0

[B158] Şandor M. Opruţa C. (2012). The effects of mineral and organic fertilizers on soil respiration in a potato field. *Bull. VASVM Agric.* 69.

[B159] Savidor A. Donahoo R. S. Hurtado-Gonzales O. Land M. L. Shah M. B. Lamour K. H. (2008). Cross-species global proteomics reveals conserved and unique processes in *Phytophthora sojae* and *Phytophthora ramorum*. *Mol. Cell. Proteomics* 7 1501–1516. 10.1074/mcp.M700431-MCP200 18316789 PMC2500229

[B160] Schornack S. Huitema E. Cano L. M. Bozkurt T. O. Oliva R. Van Damme M. (2009). Ten things to know about oomycete effectors. *Mol. Plant Pathol.* 10 795–803. 10.1111/j.1364-3703.2009.00593.x 19849785 PMC6640533

[B161] Scott P. Bader M. K. F. Williams N. M. (2016). Foliar phosphite application has minor phytotoxic impacts across a diverse range of conifers and woody angiosperms. *Physiol. Plant.* 158 124–134. 10.1111/ppl.12442 26968132

[B162] Scott P. Barber P. Hardy G. S. J. (2015). Novel phosphite and nutrient application to control *Phytophthora cinnamomi* disease. *Australas. Plant Pathol.* 44 431–436. 10.1007/s13313-015-0365-4

[B163] Schroetter S. Angeles-Wedler D. Kreuzig R. Schnug E. (2006). Effects of phosphite on phosphorus supply and growth of corn (*Zea mays*). *Landbauforschung Volkenrode* 56:87.

[B164] Shafique H. A. Sultana V. Ehteshamul-Haque S. Athar M. (2016). Management of soil-borne diseases of organic vegetables. *J. Plant Prot. Res.* 56 221–230. 10.1515/jppr-2016-0043

[B165] Shearer B. Crane C. (2009). Influence of site and rate of low-volume aerial phosphite spray on lesion development of *Phytophthora cinnamomi* and phosphite persistence in *Lambertia inermis* var. inermis and *Banksia grandis*. *Australas. Plant Pathol.* 38 288–304. 10.1071/AP09005

[B166] Shearer B. Crane C. (2012). Variation within the genus Lambertia in efficacy of low-volume aerial phosphite spray for control of Phytophthora cinnamomi. *Australas. Plant Pathol.* 41 47–57. 10.1007/s13313-011-0088-0

[B167] Shearer B. Fairman R. (2007). A stem injection of phosphite protects Banksia species and *Eucalyptus marginata* from *Phytophthora cinnamomi* for at least four years. *Australas. Plant Pathol.* 36 78–86. 10.1071/AP06085

[B168] Shearer B. Fairman R. Grant M. (2006). Effective concentration of phosphite in controlling *Phytophthora cinnamomi* following stem injection of Banksia species and *Eucalyptus marginata*. *For. Pathol.* 36 119–135. 10.1111/j.1439-0329.2006.00440.x

[B169] Silva O. Santos H. Dalla Pria M. May-De Mio L. (2011). Potassium phosphite for control of downy mildew of soybean. *Crop Prot.* 30 598–604. 10.1016/j.cropro.2011.02.015

[B170] Singh B. N. Singh B. R. Sarma B. Singh H. (2009). Potential chemoprevention of N-nitrosodiethylamine-induced hepatocarcinogenesis by polyphenolics from *Acacia nilotica* bark. *Chem. Biol. Interact.* 181 20–28. 10.1016/j.cbi.2009.05.007 19446540

[B171] Smillie R. Grant B. Guest D. (1989). The mode of action of phosphite: evidence for both direct and indirect modes of action on three *Phytophthora* spp. in plants. *Phytopathology* 79 921–926. 10.1094/PHYTO-79-921

[B172] Sousa M. F. Façanha A. R. Tavares R. M. Lino-Neto T. Gerós H. (2007). Phosphate transport by proteoid roots of Hakea sericea. *Plant Sci.* 173 550–558. 10.1016/j.plantsci.2007.08.006

[B173] Speiser B. Berner A. Häseli A. Tamm L. (2000). Control of downy mildew of grapevine with potassium phosphonate: effectivity and phosphonate residues in wine. *Biol. Agric. Hortic.* 17 305–312. 10.1080/01448765.2000.9754851

[B174] Sukarno N. Smith F. Scott E. Jones G. Smith S. (1998). The effect of fungicides on vesicular–arbuscular mycorrhizal symbiosis. III. the influence of VA mycorrhiza on phytotoxic effects following application of fosetyl-Al and phosphonate. *New Phytol.* 139 321–330. 10.1046/j.1469-8137.1998.00204.x

[B175] Sun L. R. Wang Y. B. He S. B. Hao F. S. (2018). Mechanisms for abscisic acid inhibition of primary root growth. *Plant Signal. Behav.* 13:e1500069. 10.1080/15592324.2018.1500069 30081737 PMC6204825

[B176] Tajik S. Zarinkamar F. Soltani B. M. Nazari M. (2019). Induction of phenolic and flavonoid compounds in leaves of saffron (*Crocus sativus* L.) by salicylic acid. *Sci. Hortic.* 257:108751. 10.1016/j.scienta.2019.108751

[B177] Taylor R. J. Pasche J. S. Gudmestad N. C. (2011). Effect of application method and rate on residual efficacy of mefenoxam and phosphorous acid fungicides in the control of pink rot of potato. *Plant Dis.* 95 997–1006. 10.1094/PDIS-09-10-0694 30732101

[B178] Thakur M. Sohal B. S. (2013). Role of elicitors in inducing resistance in plants against pathogen infection: a review. *ISRN Biochem.* 2013:762412. 10.1155/2013/762412 25969762 PMC4393000

[B179] Thao H. T. B. Yamakawa T. (2009). Phosphite (phosphorous acid): fungicide, fertilizer or bio-stimulator? *Soil Sci. Plant Nutr.* 55 228–234. 10.1111/j.1747-0765.2009.00365.x

[B180] Torés Montosa J. A. (2006). Field evaluation of treatments for the control of the bacterial apical necrosis of mango (*Mangifera indica*) caused by *Pseudomonas syringae* pv. *syringae*. *Eur. J. Plant Pathol.* 116 279–288. 10.1007/s10658-006-9059-7

[B181] Torto-Alalibo T. A. Tripathy S. Smith B. M. Arredondo F. D. Zhou L. Li H. (2007). Expressed sequence tags from *Phytophthora sojae* reveal genes specific to development and infection. *Mol. Plant Microbe Interact.* 20 781–793. 10.1094/MPMI-20-7-0781 17601166

[B182] Trejo-Téllez L. I. Gómez-Merino F. C. (2018). “Phosphite as an inductor of adaptive responses to stress and stimulator of better plant performance,” in *Biotic and Abiotic Stress Tolerance in Plants*, ed. Vats S. (Berlin: Springer). 10.1007/978-981-10-9029-5_8

[B183] Turner B. L. Papházy M. J. Haygarth P. M. Mckelvie I. D. (2002). Inositol phosphates in the environment. *Philos. Trans. R. Soc. Lond. Ser B Biol. Sci.* 357 449–469. 10.1098/rstb.2001.0837 12028785 PMC1692967

[B184] Tyler B. M. (2007). Phytophthora sojae: root rot pathogen of soybean and model oomycete. *Mol. Plant Pathol.* 8 1–8. 10.1111/j.1364-3703.2006.00373.x 20507474

[B185] van der Hoorn R. A. Kamoun S. (2008). From guard to decoy: a new model for perception of plant pathogen effectors. *Plant Cell* 20 2009–2017. 10.1105/tpc.108.060194 18723576 PMC2553620

[B186] van der Merwe M. D. V. Kotzé J. Hall A. (1992). Effect of phosphite in avocado roots on the zoospores of *Phytophthora cinnamomi*. South African Avocado Growers’. *Assoc. Yearbook* 15 24–26.

[B187] van West P. Appiah A. A. Gow N. A. (2003). Advances in research on oomycete root pathogens. *Physiol. Mol. Plant Pathol.* 62 99–113. 10.1016/S0885-5765(03)00044-4

[B188] Varadarajan D. K. Karthikeyan A. S. Matilda P. D. Raghothama K. G. (2002). Phosphite, an analog of phosphate, suppresses the coordinated expression of genes under phosphate starvation. *Plant Physiol.* 129 1232–1240. 10.1104/pp.010835 12114577 PMC166517

[B189] Venter E. Mansoor C. V. Sibisi P. Botha A.-M. (2014). Potassium phosphate induces tolerance against the Russian wheat aphid (*Diuraphis noxia*, Homoptera: Aphididae) in wheat. *Crop Prot.* 61 43–50. 10.1016/j.cropro.2014.03.015

[B190] Vinas M. Mendez J. C. Jiménez V. M. (2020). Effect of foliar applications of phosphites on growth, nutritional status and defense responses in tomato plants. *Sci. Hortic.* 265:109200. 10.1016/j.scienta.2020.109200

[B191] Vogel H. Thompson J. Shockman G. (1970). “Characteristic metabolic patterns of prokaryotes and eukaryotes,” in *Proceedings of the Society for General Microbiology Symposia*, (Cambridge: Cambridge Universirty).

[B192] Walker C. A. van West P. (2007). Zoospore development in the oomycetes. *Fungal Biol. Rev.* 21 10–18. 10.1016/j.fbr.2007.02.001

[B193] Walters D. Bingham I. (2007). Influence of nutrition on disease development caused by fungal pathogens: implications for plant disease control. *Ann. Appl. Biol.* 151 307–324. 10.1111/j.1744-7348.2007.00176.x

[B194] Wang-Pruski G. Coffin R. H. Peters R. D. Al-Mughrabi K. I. Platt H. W. Pinto D. (2010). Phosphorous acid for late blight suppression in potato leaves. *Am. J. Plant Sci. Biotechnol.* 4 25–29.

[B195] Wilkinson C. Holmes J. Tynan K. Colquhoun I. Mccomb J. Hardy G. S. J. (2001). Ability of phosphite applied in a glasshouse trial to control Phytophthora cinnamomi in five plant species native to Western Australia. *Australas. Plant Pathol.* 30 343–351. 10.1071/AP01055

[B196] Win J. Morgan W. Bos J. Krasileva K. V. Cano L. M. Chaparro-Garcia A. (2007). Adaptive evolution has targeted the C-terminal domain of the RXLR effectors of plant pathogenic oomycetes. *Plant Cell* 19 2349–2369. 10.1105/tpc.107.051037 17675403 PMC2002621

[B197] Wu L. Gao X. Xia F. Joshi J. Borza T. Wang-Pruski G. (2019). Biostimulant and fungicidal effects of phosphite assessed by GC-TOF-MS analysis of potato leaf metabolome. *Physiol. Mol. Plant Pathol.* 106 49–56. 10.1016/j.pmpp.2018.12.001

[B198] Xi Y. Han X. Zhang Z. Joshi J. Borza T. Aqa M. M. (2020). Exogenous phosphite application alleviates the adverse effects of heat stress and improves thermotolerance of potato (*Solanum tuberosum* L.) seedlings. *Ecotoxicol. Environ. Saf.* 190:110048. 10.1016/j.ecoenv.2019.110048 31837570

